# Inhibitory Neural Regulation of the Ca^*2*+^ Transients in Intramuscular Interstitial Cells of Cajal in the Small Intestine

**DOI:** 10.3389/fphys.2018.00328

**Published:** 2018-04-09

**Authors:** Salah A. Baker, Bernard T. Drumm, Caroline A. Cobine, Kathleen D. Keef, Kenton M. Sanders

**Affiliations:** Department of Physiology and Cell Biology, University of Nevada, Reno, NV, United States

**Keywords:** enteric nervous system, SIP syncytium, Ca^2+^ imaging, nitric oxide, gastrointestinal motility, tonic inhibition, VIP

## Abstract

Gastrointestinal motility is coordinated by enteric neurons. Both inhibitory and excitatory motor neurons innervate the syncytium consisting of smooth muscle cells (SMCs) interstitial cells of Cajal (ICC) and PDGFRα^+^ cells (SIP syncytium). Confocal imaging of mouse small intestines from animals expressing GCaMP3 in ICC were used to investigate inhibitory neural regulation of ICC in the deep muscular plexus (ICC-DMP). We hypothesized that Ca^2+^ signaling in ICC-DMP can be modulated by inhibitory enteric neural input. ICC-DMP lie in close proximity to the varicosities of motor neurons and generate ongoing Ca^2+^ transients that underlie activation of Ca^2+^-dependent Cl^−^ channels and regulate the excitability of SMCs in the SIP syncytium. Electrical field stimulation (EFS) caused inhibition of Ca^2+^ for the first 2–3 s of stimulation, and then Ca^2+^ transients escaped from inhibition. The NO donor (DEA-NONOate) inhibited Ca^2+^ transients and Nω-Nitro-L-arginine (L-NNA) or a guanylate cyclase inhibitor (ODQ) blocked inhibition induced by EFS. Purinergic neurotransmission did not affect Ca^2+^ transients in ICC-DMP. Purinergic neurotransmission elicits hyperpolarization of the SIP syncytium by activation of K^+^ channels in PDGFRα^+^ cells. Generalized hyperpolarization of SIP cells by pinacidil (K_ATP_ agonist) or MRS2365 (P2Y1 agonist) also had no effect on Ca^2+^ transients in ICC-DMP. Peptidergic transmitter receptors (VIP and PACAP) are expressed in ICC and can modulate ICC-DMP Ca^2+^ transients. In summary Ca^2+^ transients in ICC-DMP are blocked by enteric inhibitory neurotransmission. ICC-DMP lack a voltage-dependent mechanism for regulating Ca^2+^ release, and this protects Ca^2+^ handling in ICC-DMP from membrane potential changes in other SIP cells.

## Introduction

In the gastrointestinal tract, muscle bundles making up the *tunica muscularis* have intrinsic mechanisms of excitability, and this has been described as myogenic activity. In fact this level of motor control is due not only to the functions of smooth muscle cells (SMCs), because the behavior of SMCs is modulated by interstitial cells [e.g., interstitial cells of Cajal (ICC) and cells labeled with antibodies to platelet-derived growth factor receptor alpha (aka PDGFRα^+^ cells)]. ICC and PDGFRα^+^ cells are electrically coupled to SMCs (Zhou and Komuro, 1992a; Torihashi et al., 1993; Seki and Komuro, 1998; Horiguchi and Komuro, 2000), and the resulting cellular network has been referred to as the SIP syncytium (Sanders et al., 2012). Conductance changes in one type of SIP cell causes changes in the membrane potentials and excitability of coupled cells. The SIP syncytium is innervated by enteric motor neurons, and each cell type expresses receptors that can bind to and transduce inputs from neurotransmitters released from motor neurons (Chen et al., 2007; Lee et al., 2017). Neural inputs are integrated by the SIP syncytium and the output sets the moment-to-moment excitability of the SMCs, generating the underlying basis for motility patterns such as phasic contractions, summation of phasic contractions to generate tone, peristalsis and segmentation.

ICC are present in all smooth muscle portions of the GI tract, and in the small intestine there are at least 2 populations of these cells. ICC in the myenteric plexus region (ICC-MY) generate pacemaker activity that develops into electrical slow waves (Langton et al., 1989; Ward et al., 1994; Huizinga et al., 1995; Ordog et al., 1999; Sanders et al., 2014b; Drumm et al., 2017). ICC within the deep muscular plexus region (ICC-DMP) are in close contact with varicosities of excitatory and inhibitory enteric motor neurons (Rumessen et al., 1992; Zhou and Komuro, 1992b; Blair et al., 2012), express receptors that can bind to major enteric motor neurotransmitters (Chen et al., 2007), and, as above, are electrically coupled to SMCs via gap junctions (Daniel et al., 1998; Seki and Komuro, 2001). These properties of ICC-DMP led to the suggestion that they may be innervated and involved in generating post-junctional responses to motor neurotransmission. In other regions of the GI tract loss of intramuscular ICC caused changes or disruption in normal motor neurotransmission (Burns et al., 1996; Ward et al., 2000, 2006; Wang et al., 2003a; Iino et al., 2004; Klein et al., 2013; Sanders et al., 2014a). Mounting evidence also suggests that ICC-DMP are innervated and provide at least part of the receptive field for motor neurotransmission: (i) Due to the close, synaptic-like associations between ICC-DMP and nerve varicosities, neurotransmitter concentrations could be quite high near neurotransmitter receptors (Sanders et al., 2010; Bhetwal et al., 2013); (ii) functional immunohistochemistry has shown translocation of signaling molecules in ICC-DMP consistent with binding of muscarinic and NK1 receptors (Wang et al., 2003b; Iino et al., 2004); and (iii) a conductance unique to ICC-DMP (Ano1) is activated by motor neurotransmission (Zhu et al., 2011).

The precise mechanisms through which ICC transduce inputs from motor neurons are poorly understood, largely because past studies have relied upon *in vitro* experiments on isolated cells (in many cases on cells studied after several days in culture (Koh et al., 2000; D'antonio et al., 2009; So et al., 2009; Kim et al., 2012), studies on intact muscles utilizing techniques requiring fixation of tissues (Wang et al., 2003b; Iino et al., 2004), or studies using membrane permeable Ca^2+^ sensors that load to varying degrees into all cells in tissues and provide, as a result, confusing and possibly misleading information about the Ca^2+^ signaling in ICC (Huizinga et al., 2014; Zhu et al., 2016). Ca^2+^ signaling, however, is important because a major conductance in ICC-DMP that is affected by neurotransmission is a Ca^2+^-activated Cl^−^ conductance (Ano1; Zhu et al., 2011). We hypothesize that modulation of Ca^2+^ transients in ICC constitutes a major mechanism regulated by enteric neurotransmission. Therefore, we have used optogenetics and mice expressing a genetically-encoded Ca^2+^ sensor (GCaMP3) expressed specifically in murine ICC to investigate the responses of ICC-DMP to enteric inhibitory neurotransmission. Our results show that enteric inhibitory neurotransmitters exert powerful inhibitory effects on Ca^2+^ release, which would be expected to reduce activation of Ano1 and development of spontaneous transient inward currents (STICs) and reduce the excitatory drive exerted upon the SIP syncytium by ICC-DMP.

## Methods

### Animals

GCaMP3-floxed mice (B6.129S-*Gt(ROSA)26Sor*^*tm*38(*CAG*−*GCaMP*3)*Hze*^/J) and their corresponding wild-type siblings (C57BL/6) were purchased from Jackson Laboratories (Bar Harbor, MN, USA) and subsequently crossed with Kit-Cre mice (c-Kit^+/Cre−ERT2^) provided by Dr. Dieter Saur (Technical University Munich, Munich, Germany). Kit-Cre-GCaMP3 mice underwent treatment with tamoxifen at 6–8 weeks of age (2 mg for 3 consecutive days), as previously described (Baker et al., 2016), to induce activation of Cre recombinase in ICC and activate expression of GCaMP3. After tamoxifen (15 days); Kit-Cre-GCaMP3 mice were anesthetized by isoflurane inhalation (Baxter, Deerfield, IL, USA) and killed by cervical dislocation. All animals used for these experiments were handled in accordance with the National Institutes of Health Guide for the Care and Use of Laboratory Animals and approved by the Institutional Animal Use and Care Committee at the University of Nevada, Reno [Animal assurance # D16-00311 (A3500-01)].

### Tissue preparation

Following an abdominal incision, 2 cm segments of jejunum were removed and bathed in Krebs-Ringer bicarbonate solution (KRB). The jejunal segments opened along the mesenteric border, and intra-luminal contents were removed by washing with KRB. Mucosal and sub-mucosal layers were removed by sharp dissection, and the remaining *tunica muscularis* was pinned out in a Sylgard coated dish.

### Drugs and solutions

Tissues were maintained by perfusing with KRB containing (mmol/L): NaCl, 120.35; KCl, 5.9; NaHCO_3_, 15.5; NaH_2_PO_4_, 1.2; MgCl_2_, 1.2; CaCl_2_, 2.5; and glucose, 11.5. The KRB was bubbled with a mixture of 97% O_2_–3% CO_2_ and warmed to 37 ± 0.2°C. All drugs were purchased from Tocris Bioscience (Ellisville, Missouri, USA) and dissolved in solvents recommended by the manufacturer to create appropriate stock solutions. Final concentration used for experiments were obtained by diluting with KRB. All work was performed according to biosafety level II regulations.

### Responses of ICC to intrinsic nerve stimulation

Neural responses were elicited by electrical field stimulation (EFS; 1–20 Hz, 0.5 ms pulse duration; 10–15 v; 5 s trains) generated by a Grass stimulator Grass S48 stimulator (Quincy, MA, USA) and delivered via two platinum electrodes placed on either side of muscle strips. Responses evoked by EFS were completely abolished by tetrodotoxin (TTX: 1 μM, data not shown).

### Fluorescence activated cell sorting (FACS), RNA extraction, and quantitative PCR

Jejunal ICC were dispersed from Kit^+/copGFP^ mice as previously described (Zhu et al., 2009). Enriched populations of ICC were sorted by FACS (FACSAria II; Becton-Dickinson) using an excitation laser (488 nm) and emission filter (530/30 nm). Sorting was performed using a 130-μm nozzle and a sheath pressure of 12 psi. RNA was prepared from sorted ICC and dispersed unsorted jejunal cells of the *tunica muscularis* before sorting using an illustra RNAspin Mini RNA Isolation Kit (GE Healthcare). The PCR primers used and their GenBank accession numbers are listed in Table 1. Quantitative PCR (qPCR) was performed using SYBR green chemistry on the 7500 HT Real-time PCR System (Applied Biosystems) and analyzed, as previously described (Baker et al., 2016).

**Table 1:** Summary table of sGC, PKG, IRAK, VIP, and PACAP receptor primer sequences.

**Gene**	**Sequence**	**GenBank accession number**
*mGapdh-F*	AGACGGCCGCATCTTCTT	NM_008084
*mGapdh-R*	TTCACACCGACCTTCACCAT	
*mGucy1a1-F*	GTTGTCGGAGTGAAGATGCC	NM_021896
*mGucy1a1-R*	TGATCTCGGGGTGAACACAA	
*mGucy1b1-F*	GATCCGCAATTATGGTCCCG	NM_017090
*mGucy1b1-R*	AACATCTGCAGGATTTCGCC	
Mrvi1-F	TCAGGATTGGAGAGGGTGGT	NM_001177973
Mrvi1-R	GGGTGACGAAACCTTGATAGC	
Vipr1-F	TCAATGGCGAGGTGCAGGCAG	NM_011703
Vipr1-R	TGTGTGCTGCACGAGACGCC	
Vipr2-F	AGGAAGCTGCACTGCACAAGGAA	NM_009511
Vipr2-R	GAGCTTGCAGCCAACCCAGGA	
Adcyap1r1-F	AACGACCTGATGGGCCTAAA	NM_007407
Adcyap1r1-R	TGTCATCCAGACTTGGTCCG	
Prkg1-F	TATCATCAGGCAGGGTGCAA	NM_011160
Prkg1-R	GACAGCTTCTGCGGCAATAA	

*Table lists sGC (Gucy1a1, Gucy1b1), PKG (Prkg1), IRAK (Mrvi1), VIP receptors (Vipr1, Vipr2), and PACAP receptor (Adcyap1r1) genes used in this study including their name, primer sequences and gene bank accession numbers*.

### Calcium imaging

For imaging studies, the muscles were equilibrated with continuous perfusion of warmed KRB solution at 37°C for 1 h. Imaging was performed with a spinning-disk confocal microscope (CSU-W1 spinning disk; Yokogawa Electric Corporation) mounted to an upright Nikon Eclipse FN1 microscope equipped with a 60x 1.0 NA CFI Fluor lens (Nikon instruments INC, NY, USA). GCaMP3, expressed in ICC within the jejunal muscles, was excited at 488 nm using a laser coupled to a Borealis system (ANDOR Technology, Belfast, UK). Emitted fluorescence (>515 nm) was captured using a high-speed EMCCD Camera (Andor iXon Ultra; ANDOR Technology, Belfast, UK). Image sequences were acquired at 33 fps using MetaMorph software (Molecular Devices Inc., CA, USA). In some experiments images were acquired with an Eclipse E600FN microscope (Nikon Inc., Melville, NY, USA) equipped with a 60x 1.0 CFI Fluor lens (Nikon instruments Inc., NY, USA). In this system, GCaMP3 was excited at 488 nm (T.I.L.L. Polychrome IV, Grafelfing, Germany), as previously described (Baker et al., 2013). All Ca^2+^ imaging experiments were performed in the presence of nicardipine (100 nM) to minimize movement artifacts resulting from contractions.

### Calcium event analysis

Analysis of Ca^2+^ activity in ICC-DMP was performed, as described previously (Baker et al., 2016). Briefly, movies of Ca^2+^ activity in ICC-DMP were converted to a stack of TIFF (tagged image file format) images and imported into custom software (Volumetry G8c, GW Hennig) for initial pre-processing analysis. Tissue movement was stabilized to ensure accurate measurement of Ca^2+^ transients from ICC-DMP. Whole cell ROIs were used to generate spatio-temporal (ST) maps of Ca^2+^ activity in individual ICC-DMP recorded *in situ*. ST maps were then imported as TIFF files into Image J (version1.40, National Institutes of Health, MD, USA, http://rsbweb.nih.gov/ij) for *post-hoc* quantification analysis of Ca^2+^ events.

### Statistics

Ca^2+^ event frequency in ICC-DMP was expressed as the number of events fired per cell per second (s^−1^). Ca^2+^ event amplitude was expressed as ΔF/F_0_, the duration of Ca^2+^ events was expressed as full duration at half maximum amplitude (FDHM), and Ca^2+^ event spatial spread was expressed as μm of cell propagated per Ca^2+^ event. Unless otherwise stated, data is represented as mean ± standard error (S.E.M.). Statistical analysis was performed using either a student's *t*-test or with an ANOVA test where appropriate (data was tested for normality using a D'Agostino-Pearson omnibus normality test). In all statistical analyses, *P* < 0.05 was taken as significant. *P* < 0.05 are represented by a single asterisk (^*^), *P* < 0.01 are represented by two asterisks (^**^), *P* < 0.001 are represented by three asterisks (^***^) and *P* < 0.0001 are represented by four asterisks (^****^). When describing data throughout the text, *n* refers to the number of animals used in that dataset while *c* refers to the numbers of cells used in that same data set.

## Results

### Enteric nerve stimulation produces inhibition of Ca^2+^ transients in ICC-DMP

**Figure 1:**
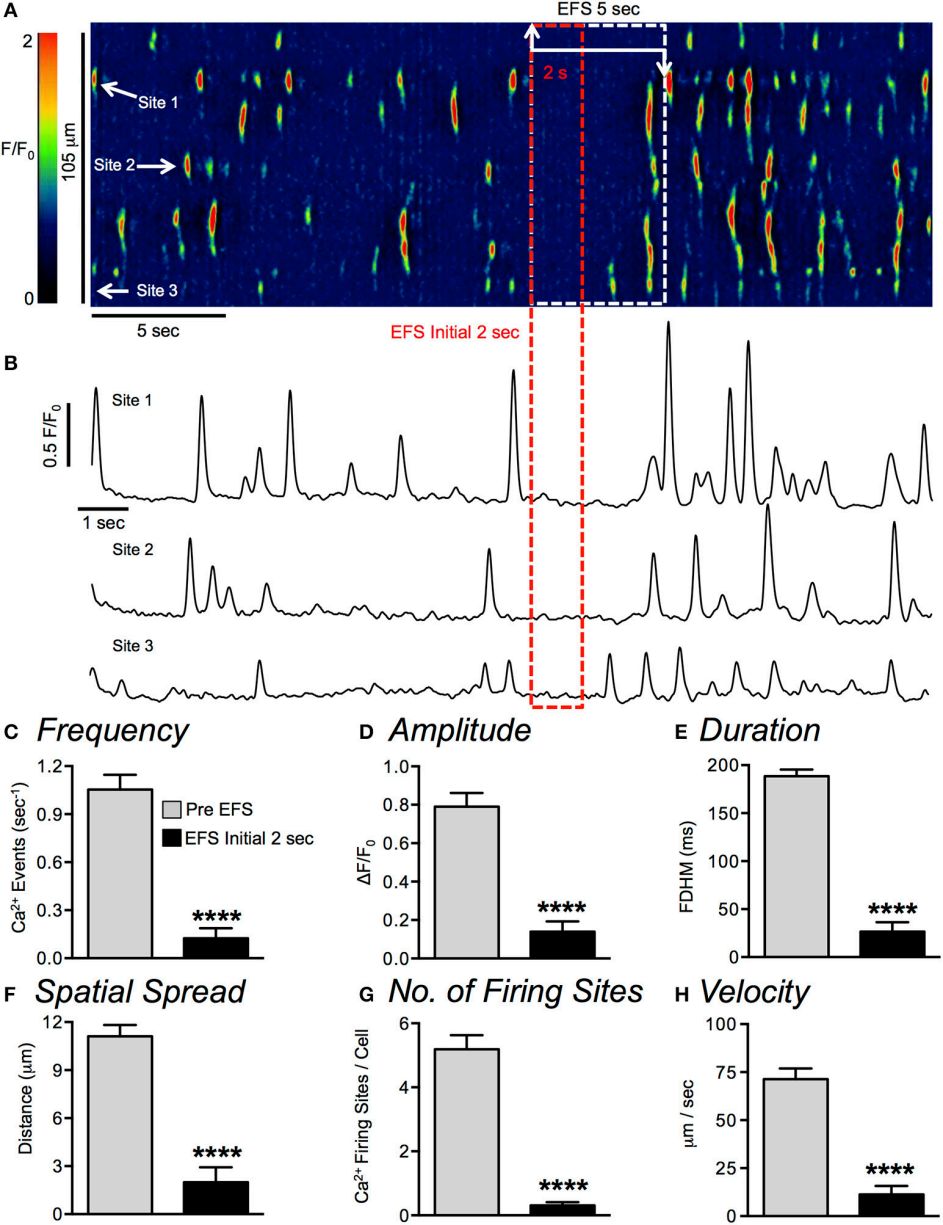
Ca^2+^ transients in ICC-DMP are inhibited after initiation of nerve stimulation. **(A)** Representative ST map of Ca^2+^ transients in a single ICC-DMP taken from a recording *in situ* (60x objective). A color-coded overlay and calibration scale was imported to depict fluorescence intensity (*F*/*F*_0_) and enhance visualization of Ca^2+^ sites. Low fluorescence areas are indicated in dark blue or black. High intensity fluorescence areas are indicated in red and orange. EFS (10 Hz, for 5 s) was applied, as indicated by the white dashed box. The red dashed box highlights the initial 2 s of EFS. The white arrows near the beginning of the ST map indicate 3 specific Ca^2+^ firing sites and their firing activity is plotted in the traces shown in **(B)**. **(C–H)** Summary data quantifying the effects of nerve evoked responses on ICC-DMP Ca^2+^ transient frequency **(C)**, amplitude **(D)**, duration **(E)**, spatial spread **(F)**, number of Ca^2+^ firing sites **(G)** and Ca^2+^ transient velocity **(H)** during the initial 2 s of EFS (*n* = 19, *c* = 48). All statistical analyses are in comparison to the pre-EFS period. ^****^P < 0.0001.

Ca^2+^ transients in ICC-DMP were ongoing and stochastic in nature, as previously reported (Baker et al., 2016). In the absence of stimulation there was no evidence of coordination between the events occurring within single cells or in other cells within a field-of-vision (FOV), suggesting there was no voltage-dependent regulation of Ca^2+^ transients in ICC-DMP. Electrical field stimulation (EFS; 10 Hz, 0.5 ms for 5 s trains) resulted in multiphasic responses (inhibition of Ca^2+^ transients followed by enhancement of these events), as shown in a representative spatiotemporal map (ST map) and traces of Ca^2+^ transients (Figures 1A,B). Prior to EFS, Ca^2+^ transients fired from multiple sites (representative sites marked with white arrows along the vertical axis of the ST map). During EFS (5 s), an initial inhibitory period was observed in which Ca^2+^ transients ceased for ~2 s. The firing of Ca^2+^ transients escaped from inhibition during the final 3 s of EFS, and a robust increase in Ca^2+^ transient firing was apparent during this period and after cessation of EFS (Figures 1A,B).

In this study we focused our analysis on the inhibitory responses imposed on ICC-DMP by nerve stimulation, so Ca^2+^ transients during the initial 2 s of EFS were analyzed (Figures 1C–H). During the inhibitory phase, Ca^2+^ transients decreased from 1.05 ± 0.09 events s^−1^ before EFS to 0.125 ± 0.06 events s^−1^ during stimulation (Figure 1C, *n* = 19, *c* = 48, *P* < 0.0001). The amplitude of Ca^2+^ transients decreased from 0.8 ± 0.07 ΔF/F_0_ prior to EFS to 0.14 ± 0.05 ΔF/F_0_ during the inhibitory phase (Figure 1D, *n* = 19, *c* = 48, *P* < 0.0001), duration decreased from 189 ± 7 ms before EFS to 27 ± 9.7 ms (Figure 1E, *n* = 19, *c* = 48, *P* < 0.0001), and the spatial spread of Ca^2+^ transients decreased from 11.1 ± 0.7 to 2 ± 0.9 μm (Figure 1F, *n* = 19, *c* = 48, *P* < 0.0001). The number of active firing sites also decreased during the inhibitory phase of EFS from 5.2 ± 0.4 to 0.3 ± 0.09 (Figure 1G, *n* = 19, *c* = 48, *P* < 0.0001). Lastly, the propagation velocity of Ca^2+^ signals decreased from 71.4 ± 5.6 μm/s prior EFS to 11.4 ± 4.4 μm/s during the first 2 s of EFS (Figure 1H, *n* = 19, *c* = 48, *P* < 0.0001).

### Ca^2+^ firing sites in ICC-DMP have variable escape from inhibition characteristics

**Figure 2:**
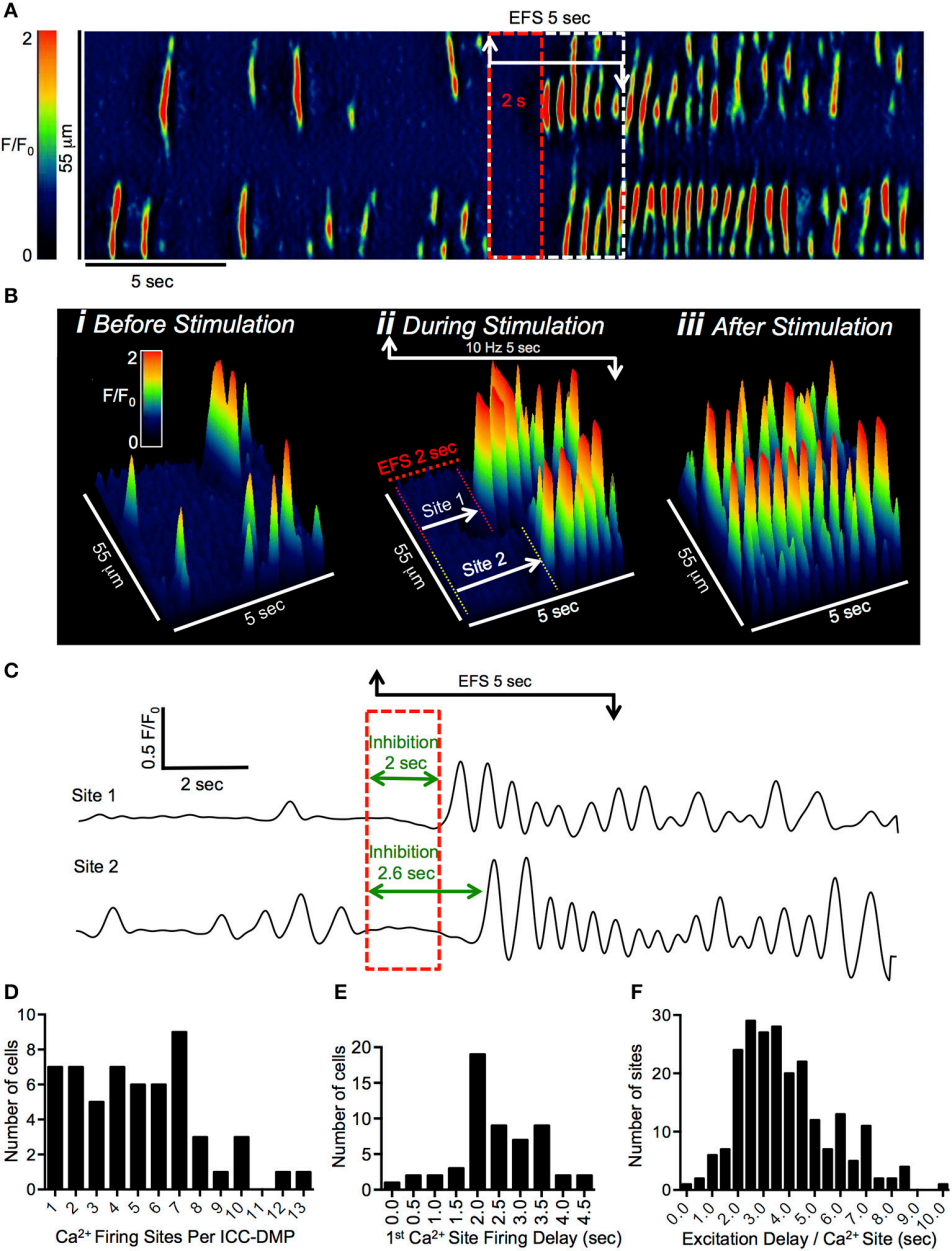
Escape from EFS inhibition occurs at variable times at diferent Ca^2+^ firing sites in ICC-DMP. **(A)** Representative ST map of Ca^2+^ transients in a single ICC-DMP *in situ*. The period of EFS (10 Hz; 5 s) is indicated by the white dashed box, and the red dashed box highlights the initial 2 s of EFS. **(B)** 3-D plots illustrating Ca^2+^ transient firing in the ICC-DMP shown in **(A)** in the 5 s pre-EFS **(i)**, during EFS **(ii)**, and post-EFS **(iii)**. The white arrows in **(Bii)** highlight 2 distinct firing sites, which were inhibited during the initial phase of EFS and then escaped inhibition at different times. The durations of inhibition at site 1 and 2 are highlighted by the red and yellow dashed lines, respectively, and the activities of the 2 sites are plotted in **(C)**. The initial 2 s of EFS is indicated by the red dashed box and green lines indicate different inhibition times for each site. **(D)** Summary histogram showing the number of Ca^2+^ firing sites contained in ICC-DMP (*n* = 19, *c* = 48). **(E)** Summary histogram showing the timing of the first Ca^2+^ firing site in ICC-DMP to escape from inhibition during EFS (*n* = 19, *c* = 48). **(F)** Summary histogram showing the times at which all Ca^2+^ firing sites in ICC-DMP escaped from inhibition during EFS (*n* = 19, *c* = 48).

In this and a previous study we noted a range in the number of Ca^2+^ firing sites in ICC-DMP, from a single site to 13 sites (Baker et al., 2016). In the present study ICC-DMP averaged 5.2 ± 0.4 firing sites per cell (Figure 2D, *n* = 19, *c* = 48). The nature of Ca^2+^ transients during sustained EFS (5 s) was examined in more detail by the analysis described in Figure 2. Similar to the previous example, Ca^2+^ transients ceased during the initial 2 s of EFS, as shown in the ST map (Figure 2A). During the final 3 s of EFS, Ca^2+^ firing sites escaped from inhibition, but each site escaped at different times, as illustrated by 3-D plots (Figure 2Bii). Ca^2+^ transients in the cell shown in Figure 2 originated from 2 distinct firing sites, as indicated by the white arrows in the 3-D plots (Figure 2Bii). The activities of these sites are also plotted as line traces in Figure 2C. Site 1 was the first site to escape from inhibition, with a ~2 s delay from the onset of EFS to the first Ca^2+^ transient that occurred at this site (Figure 2C). A greater period of inhibition was observed at site 2; a delay of ~2.6 s occurred at this site (Figure 2C). The inhibitory period from the onset of EFS to the first appearance of a Ca^2+^ transient at all firing sites in ICC-DMP averaged 2.4 ± 0.1 s (range 0.2–4.5 s; Figure 2E, *n* = 19, *c* = 48). The delay periods describing the escape from inhibition are plotted as a summary histogram in Figure 2F. The average inhibitory period at all sites was 3.8 ± 1.8 s, but ranged from 0.2 to 9.9 s (Figure 2F, *n* = 19, *c* = 48).

**Figure 3:**
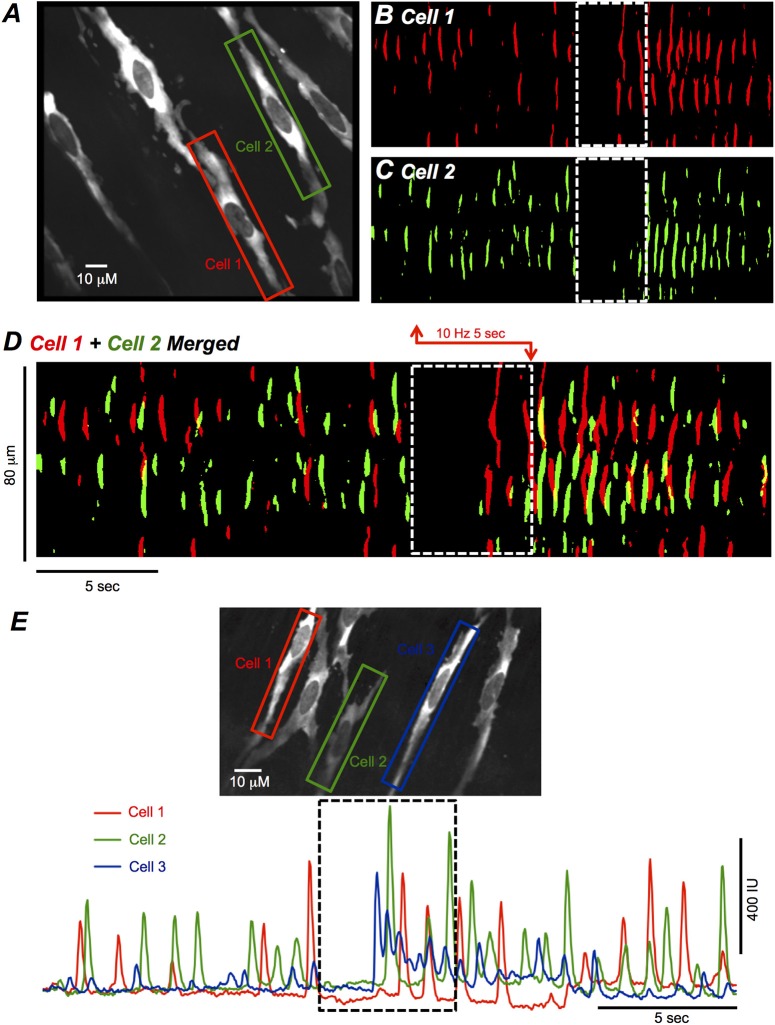
EFS effects on Ca^2+^ transients in multiple ICC-DMP. **(A)** FOV of ICC-DMP *in situ* recorded with a 60x objective showing 2 adjacent cells illustrated by the green and red ROIs. The activities of these cells are plotted as ST maps of cell 1 **(B)** and cell 2 **(C)** represented in **(A)** which have been uniformly colored to show all Ca^2+^ activity in the cell as either red (cell 1) or green (cell 2). These ST maps are merged in **(D)**. **(E)** FOV of ICC-DMP *in situ* recorded with a 60x objective showing 3 cells illustrated by the red, green, and blue ROIs. The summated Ca^2+^ activity is plotted in the color-coded traces shown in the bottom half of the panel with the period of EFS (10 Hz, 5 s) indicated by the dashed black box. Note that all 3 cells in the FOV ceased activity at the initiation of EFS, but Ca^2+^ transients escaped from inhibition at different points in time during stimulation.

It was also apparent that different ICC-DMP within a FOV escaped inhibition at variable times and did not show a coordinated escape response. For example, a FOV containing several ICC-DMP is shown in Figure 3A. Two cells are highlighted by the red and green ROIs, and the Ca^2+^ transients in these cells are displayed in ST maps in Figures 3B,C. When these ST maps were merged, it can be seen that cell 1 and 2 escaped the inhibitory effects of EFS at different points in time, with a single Ca^2+^ firing site active in cell 2 before anything occurred in cell 1 (Figure 3D). Another example is provided in which Ca^2+^ transients in 3 ICC-DMP in a FOV were plotted in Figure 3E. Here again, the cells did not escape inhibition at the same time points. This example also illustrates the point that Ca^2+^ transients in all ICC-DMP ceased at the onset of EFS (Figures 3D,E).

### Nitrergic regulation of Ca^2+^ transients in ICC-DMP

**Figure 4:**
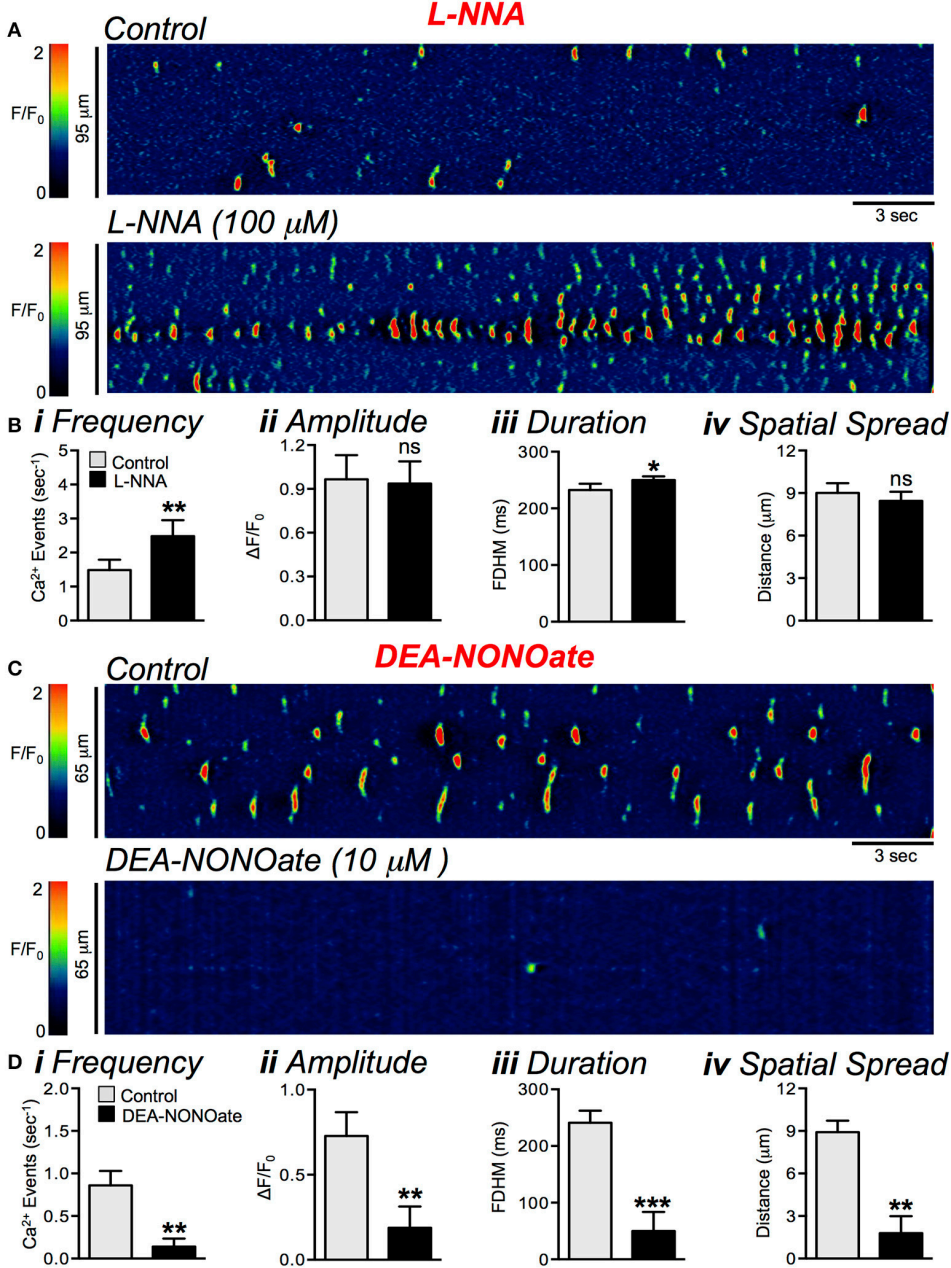
Nitrergic modulation of basal Ca^2+^ transients in ICC-DMP. **(A)** Representative ST maps showing the effects of L-NNA (100 μM) on basal Ca^2+^ transients in ICC-DMP. **(B)** Summary bar graphs showing the effects of L-NNA (100 μM) on the frequency **(i)**, amplitude **(ii)**, duration **(iii)**, and spatial spread **(iv)** of Ca^2+^ transients in ICC-DMP (*n* = 5, *c* = 11). **(C)** Representative ST maps showing the effects of DEA-NONOate (10 μM) on Ca^2+^ transients in ICC-DMP. **(D)** Summary graphs showing the effects of DEA-NONOate (10 μM) on the frequency **(i)**, amplitude **(ii)**, duration **(iii)**, and spatial spread **(iv)** of Ca^2+^ transients in ICC-DMP (*n* = 5, *c* = 10). Paired *t*-test was used; ns = *P* > 0.05, ^*^P < 0.05, ^**^P < 0.01, ^***^P < 0.001.

The nitric oxide synthase (NOS) inhibitor *N*_ω_-Nitro-L-arginine (L-NNA, 100 μM) incubated for 15 min, increased the firing frequency of Ca^2+^ transients in ICC-DMP from 1.5 ± 0.3 s^−1^ in control to 2.5 ± 0.5 s^−1^, as shown in Figures 4A,Bi (*P* = 0.0042, *n* = 5, *c* = 11). The duration of Ca^2+^ transients was also increased by L-NNA from 232 ± 11 to 250 ± 6.5 ms (Figure 4Biii, *P* = 0.04, *n* = 5, *c* = 11). However, neither the amplitude (Figure 4Bii, *P* = 0.62, *n* = 5, *c* = 11) nor the spatial spread (Figure 4Biv, *P* = 0.35, *n* = 5, *c* = 11) of Ca^2+^ transients was affected significantly by L-NNA.

The NO donor, DEA-NONOate (10 μM) caused effects opposite of L-NNA and Ca^2+^ transients were dramatically inhibited by this compound Figure 4C. In these experiments the firing frequency of Ca^2+^ transients during control conditions was 0.9 ± 0.2 s^−1^, and was reduced to 0.14 ± 0.09 s^−1^ by DEA-NONOate (Figure 4Di, *P* = 0.0014, *n* = 5, *c* = 10). The amplitude of Ca^2+^ transients was reduced from 0.7 ± 0.14 ΔF/F_0_ to 0.2 ± 0.13 ΔF/F_0_ in DEA-NONOate (Figure 4Dii, *P* = 0.0097, *n* = 5, *c* = 10), duration was decreased by DEA-NONOate from 242 ± 21.2 ms to 50 ± 33.6 ms (Figure 4Diii, *P* = 0.0003, *n* = 5, *c* = 10), and spatial spread was reduced from 8.9 ± 8 μm to 1.8 ± 1.2 μm (Figure 4Div, *P* = 0.001, *n* = 5, *c* = 10). These results suggest that NO was released tonically from enteric nerves, however at lower levels than was achieved by addition of DEA-NONOate (10 μM), and NO modulates the basal level of firing of Ca^2+^ transients in ICC-DMP.

**Figure 5:**
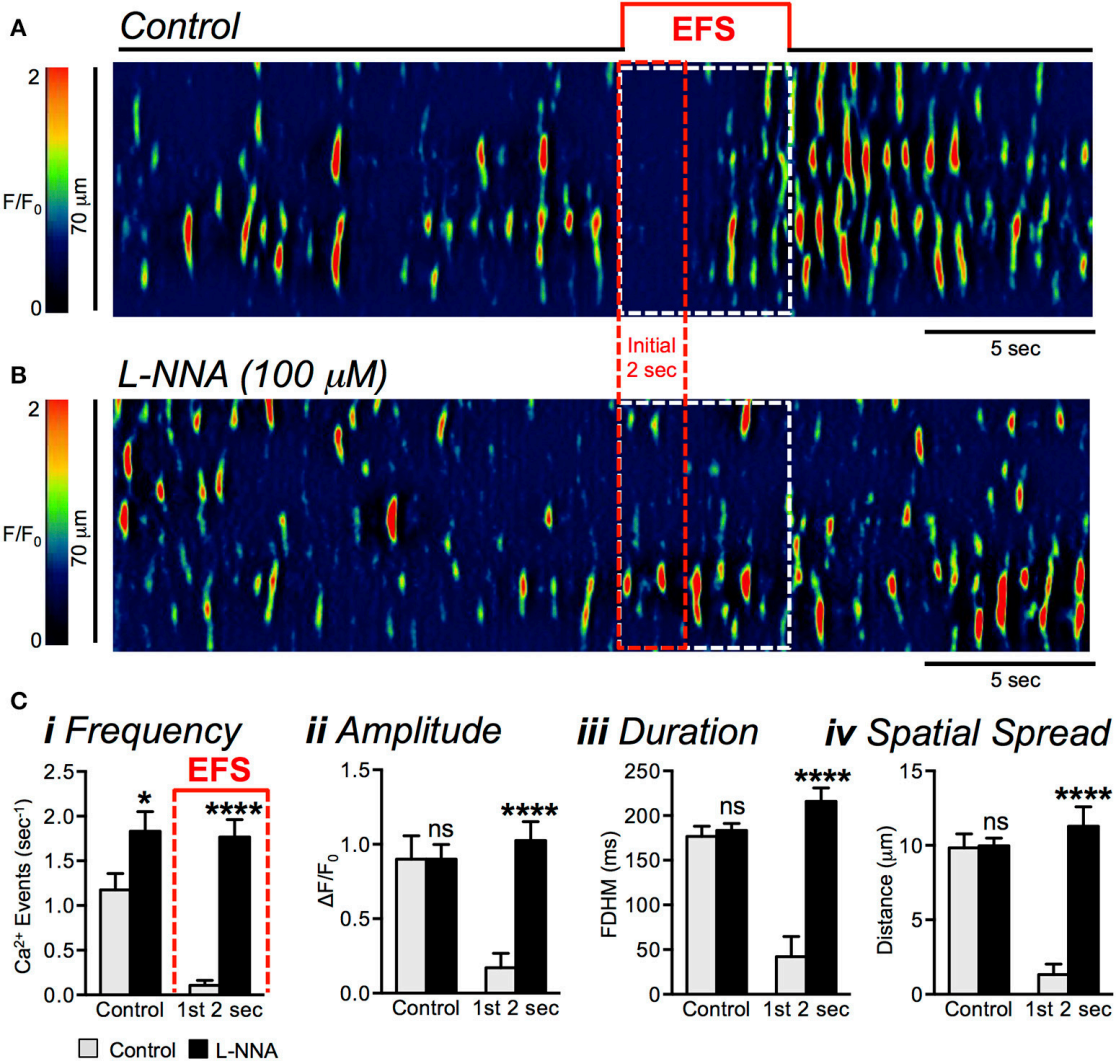
Effects of L-NNA on EFS-evoked inhibition of Ca^2+^ transients. **(A,B)** Representative ST maps of ICC-DMP showing inhibition of Ca^2+^ transients initially during sustained EFS (**A**; EFS at 10 Hz for 5 s; indicated by the solid red line and dotted white box in ST maps) and the relief of EFS evoked inhibition on Ca^2+^ transients in the presence of L-NNA (100 μM; **B**). The dotted red box indicates the initial 2 s of sustained EFS. **(C)** Summary data showing the inhibitory effects of L-NNA (100 μM) on Ca^2+^ transient frequency **(i)**, amplitude **(ii)**, duration **(iii)**, and spatial spread **(iv)** in ICC-DMP during control conditions (pre-EFS), and in the 1st 2 s of EFS (*n* = 5, *c* = 15). ns = *P* > 0.05, ^*^P < 0.05, ^****^P < 0.0001.

As above, EFS evoked inhibition of Ca^2+^ transients for about ~2 s after the onset of stimulation (Figure 5A). L-NNA (100 μM) blocked the initial inhibitory period, and Ca^2+^ transients persisted during the initial 2 s of EFS (Figure 5B). The firing frequency of Ca^2+^ transients during the first 2 s of EFS in control conditions was 0.1 ± 0.06 s^−1^, and this increased to 1.8 ± 0.2 s^−1^ in the presence of L-NNA (Figure 5Ci, *P* < 0.0001, *n* = 5, *c* = 15). L-NNA also increased the amplitude of Ca^2+^ transients from 0.2 ± 0.1 ΔF/F_0_ to 1 ± 0.1 ΔF/F_0_ (Figure 5Cii, *P* < 0.0001, *n* = 5, *c* = 15), the duration from 42 ± 14 ms to 216 ± 14.9 ms (Figure 5Ciii, *P* < 0.0001, *n* = 5, *c* = 15), and the spatial spread from 1.3 ± 0.7 μm to 11.3 ± 1.3 μm (Figure 5Civ, *P* < 0.0001, *n* = 5, *c* = 15).

### Purinergic signaling had minimal effects on ICC-DMP Ca^2+^ transients

**Figure 6:**
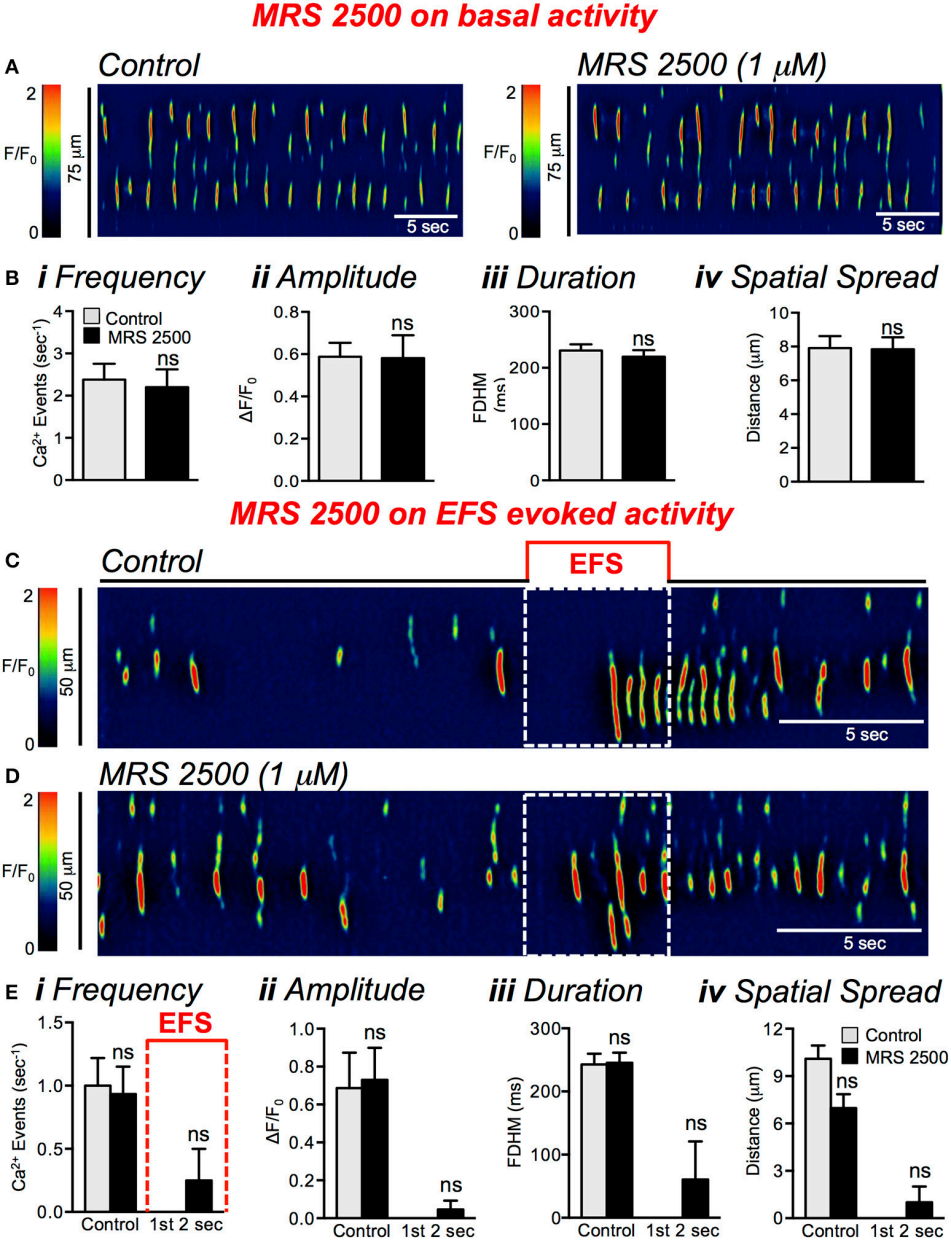
The effect of MRS 2500 on basal activity and EFS-evoked inhibitory effects on Ca^2+^ responses. **(A)** Representative ST map showing lack of effects of MRS 2500 (1 μM) on basal Ca^2+^ transients in ICC-DMP. **(B)** Summary data showing the effects of MRS 2500 (1 μM) on basal Ca^2+^ transient activity in ICC-DMP. Neither frequency **(i)**, amplitude **(ii)**, duration **(iii)**, or spatial spread **(iv)** were affected by MRS2500 (*n* = 5; *c* = 10; ns = *P* > 0.05). **(C,D)** Representative ST maps showing the effects of MRS 2500 (1 μM) on Ca^2+^ transients during EFS (10 Hz for 5 s; indicated by the red line and dotted white box in ST maps). **(E)** Summary data showing the lack of effects of MRS 2500 (1 μM) on the inhibition of Ca^2+^ transients during the first 2 s of EFS: frequency **(i)**, amplitude **(ii)**, duration **(iii)**, and spatial spread **(iv)** in ICC-DMP during control conditions (pre-EFS), and within the first 2 s of sustained EFS (*n* = 5, *c* = 6). ns = *P* > 0.05.

As several studies have reported, enteric inhibitory signaling by purines is mediated by P2Y1 receptors in post-junctional cells (Hwang et al., 2012; Gallego et al., 2014; Kito et al., 2014; Baker et al., 2015). The P2Y1 selective antagonist, MRS 2500 (1 μM), had little to no effect on basal firing of Ca^2+^ transients (Figure 6A); none of the Ca^2+^ transient parameters analyzed were changed significantly by this compound: frequency (*P* = 0.53), amplitude (*P* = 0.91), duration (*P* = 0.48), or spatial spread (*P* = 0.86) (Figures 6Bi–iv, *n* = 5, *c* = 10). MRS 2500 (1 μM) also failed to affect Ca^2+^ transients in ICC-DMP during EFS, as shown in Figures 6C–E. The amplitude, duration and spatial spread of Ca^2+^ transients were also unaffected by MRS 2500 during the initial 2s of EFS (Figure 6E, *P* = 0.36, *n* = 5, *c* = 8).

**Figure 7:**
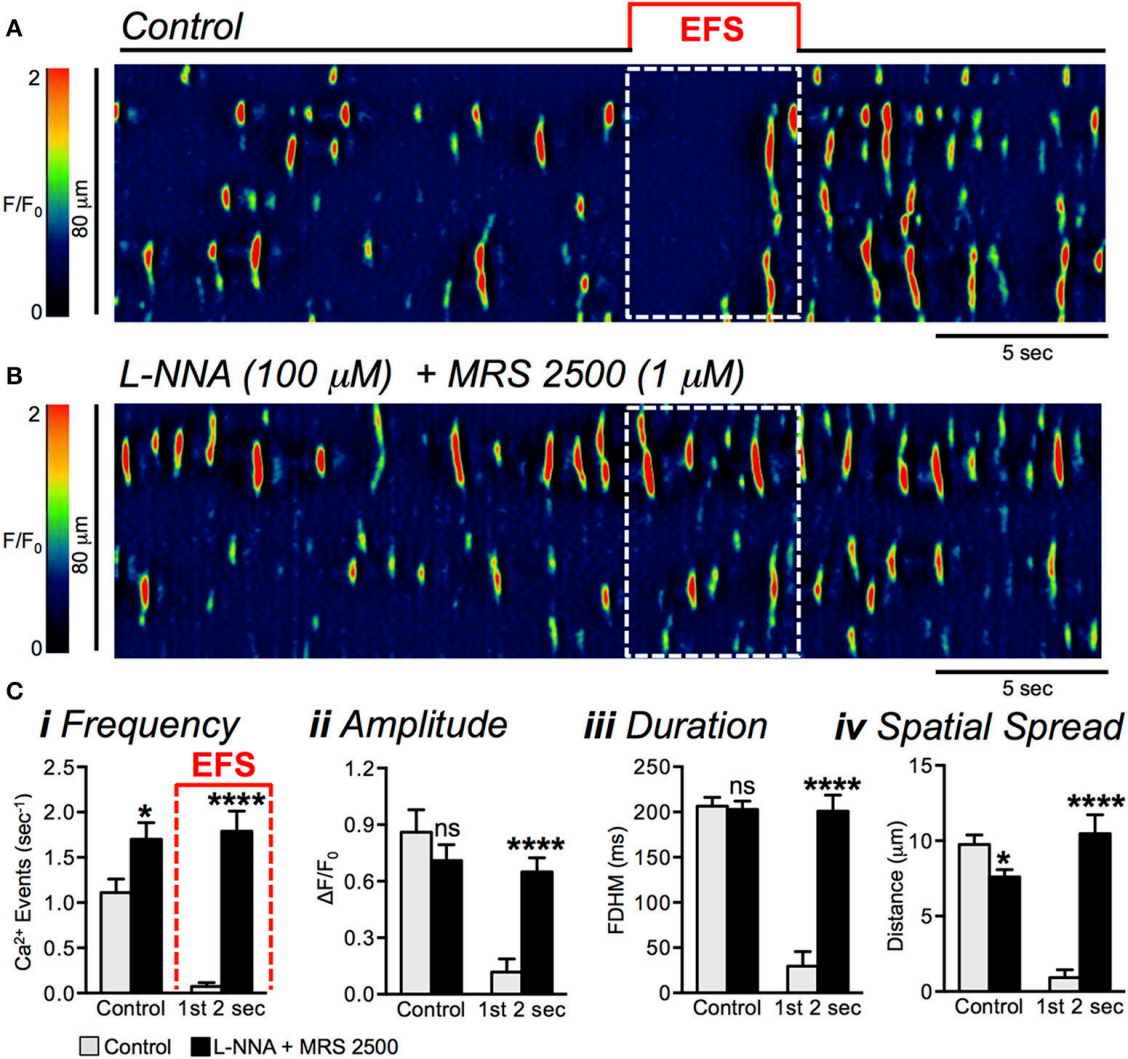
Effects of combining L-NNA + MRS 2500 on EFS-evoked inhibitory Ca^2+^ responses in ICC-DMP. **(A,B)** Representative ST maps of ICC-DMP showing inhibition of Ca^2+^ transients initially during sustained EFS **(A)**; EFS at 10 Hz for 5 s; indicated by the solid red line and dotted white box in ST maps) and the blockade of EFS evoked inhibition in response to a combination of L-NNA (100 μM) and MRS 2500 (1 μM; **B**). **(C)** Summary data showing the blockade of EFS evoked inhibitory effects by L-NNA (100 μM) + MRS 2500 (1 μM) on Ca^2+^ transient frequency **(i)**, amplitude **(ii)**, duration **(iii)**, and spatial spread **(iv)** in ICC-DMP during control conditions (pre-EFS), and in the 1st 2 s of EFS (*n* = 9, *c* = 26). ns = *P* > 0.05, ^*^P < 0.05, ^****^P < 0.0001.

While the P2Y1 receptor antagonist had no resolvable effect on the inhibition of Ca^2+^ transients in response to EFS, previous studies have found interactions between purinergic and nitrergic neurotransmitter release (Durnin et al., 2017). Therefore, we also tested the effects of combining L-NNA and MRS 2500 (Figures 7A–C). This combination blocked the inhibitory phase on Ca^2+^ transients after initiation of EFS in a manner similar to the effects of L-NNA alone. During the first 2 s after initiation of EFS Ca^2+^ transient firing frequency increased from 0.08 ± 0.04 to 1.8 ± 0.2 s^−1^ (Figure 7Ci, *P* < 0.0001, *n* = 9, *c* = 26). The amplitude of Ca^2+^ transients increased during this period from 0.1 ± 0.07 ΔF/F_0_ to 0.7 ± 0.08 ΔF/F_0_ (Figure 7Cii, *P* < 0.0001, *n* = 9, *c* = 26), Ca^2+^ transient duration increased from 30 ± 16.2 ms to 201 ± 17.83 ms (Figure 7Ciii, *P* < 0.0001, *n* = 9, *c* = 26), and the spatial spread of Ca^2+^ transients increased from 0.9 ± 0.5 μm to 10.5 ± 1.3 μm (Figure 7Civ, *P* < 0.0001, *n* = 9, *c* = 26).

### Effects of SIP syncytium hyperpolarization on Ca^2+^ transients

The purine neurotransmitter(s) in GI muscles cause significant hyperpolarization due to opening of apamin-sensitive, small conductance Ca^2+^-activated K^+^ channels (Banks et al., 1979; Matsuda et al., 2004; Gallego et al., 2008), but recent studies have shown these responses are generated by another cell-type in the SIP syncytium (Kurahashi et al., 2011, 2014). Therefore, purinergic responses conveyed to ICC-DMP would be in the form of membrane hyperpolarization. To simulate this type of response, we tested whether hyperpolarization of the SIP syncytium by MRS 2365 (acting by hyperpolarization of PDGFRα^+^ cells; Kurahashi et al., 2014) or pinacidil (acting by hyperpolarization of SMCs; Kito et al., 2005) affected Ca^2+^ transients in ICC-DMP.

**Figure 8:**
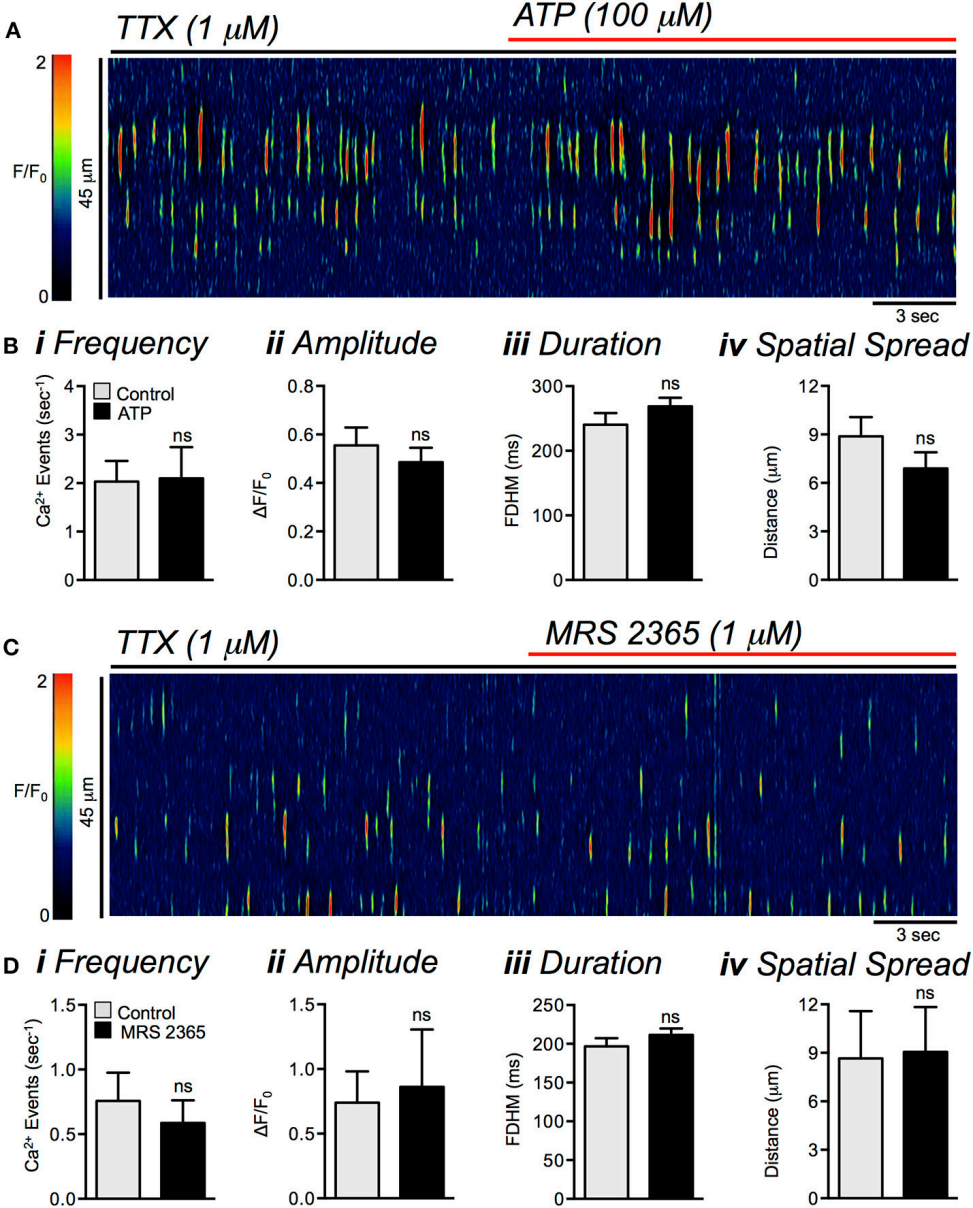
Lack of effects of purinergic agonists on Ca^2+^ transients in ICC-DMP. **(A)** Representative ST map showing lack of effects of ATP (100 μM) on Ca^2+^ transients in ICC-DMP in the presence of TTX (1 μM). **(B)** Summary bar graphs showing the lack of effects of ATP (100 μM) on the frequency **(i)**, amplitude **(ii)**, duration **(iii)**, and spatial spread **(iv)** of Ca^2+^ transients in ICC-DMP in the presence of TTX (*n* = 3, *c* = 6). ns = *P* > 0.05. **(C)** Representative ST maps showing the lack of effects of P2Y1 receptor agonist MRS 2365 (1 μM) on Ca^2+^ transients in ICC-DMP in the presence of TTX. **(D)** Summary bar graphs showing lack of the effects of MRS 2365 (1 μM) on the frequency **(i)**, amplitude **(ii)**, duration **(iii)**, and spatial spread **(iv)** of Ca^2+^ transients in ICC-DMP in the presence of TTX (*n* = 3, *c* = 5). ns = *P* > 0.05.

**Figure 9:**
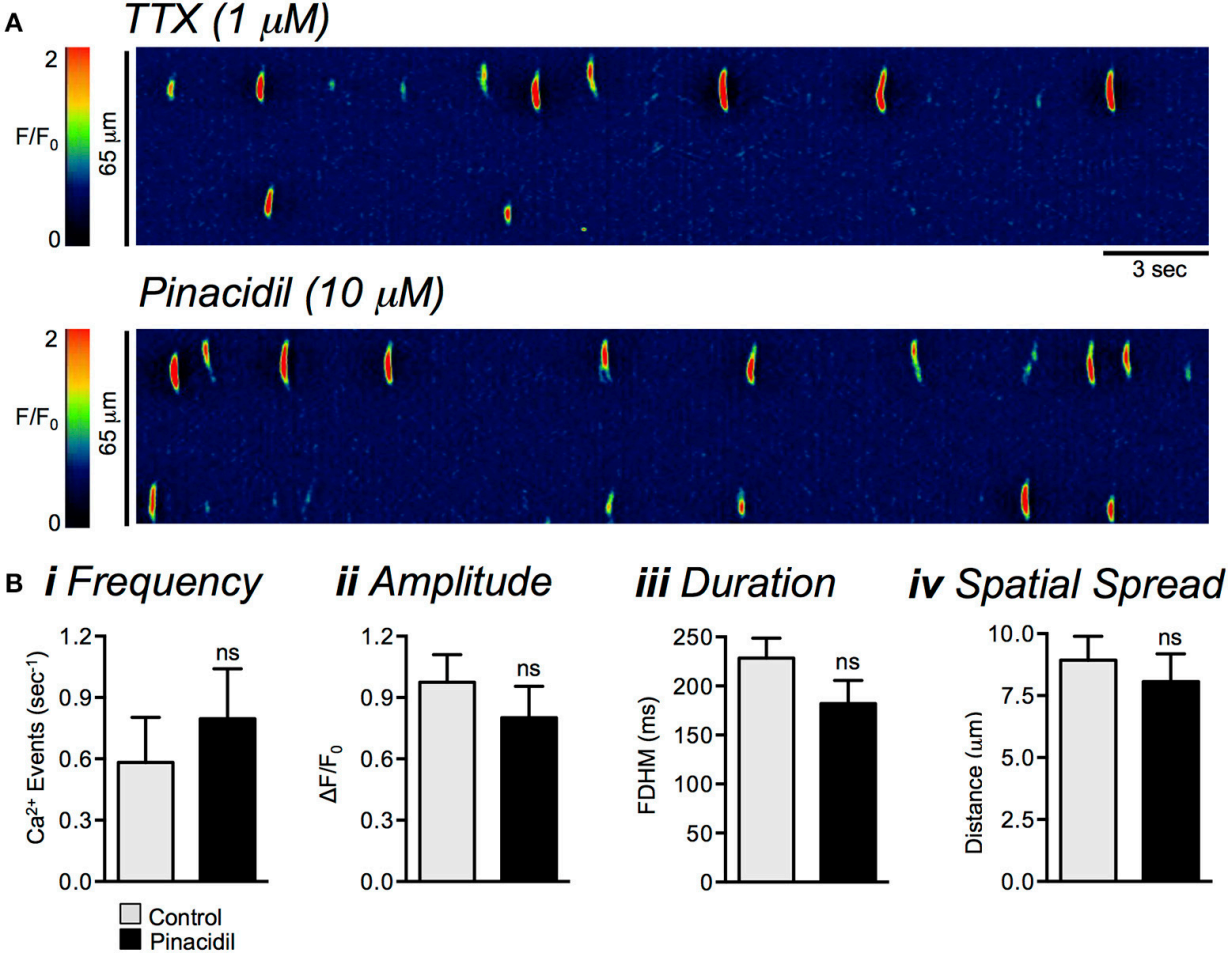
The effects of pinacidil on Ca^2+^ transients in ICC-DMP. **(A)** Representative ST maps showing the effects of pinacidil (10 μM) on Ca^2+^ transients in ICC-DMP in the presence of TTX (1 μM). **(B)** Summary bar graphs showing the effects of pinacidil (10 μM) on the frequency **(i)**, amplitude **(ii)**, duration **(iii)**, and spatial spread **(iv)** of Ca^2+^ transients in ICC-DMP in the presence of TTX (*n* = 4, *c* = 16). ns = *P* > 0.05.

Application of purinergic agonists had no significant effects on Ca^2+^ transients in ICC-DMP. In the presence of TTX, ATP (100 μM) also had no significant effects on Ca^2+^ transient frequency (*P* = 0.85), amplitude (*P* = 0.31), duration (*P* = 0.1) or spatial spread (*P* = 0.12, Figures 8A,B, *n* = 3, *c* = 6). Similarly, the selective P2Y1 receptor agonist MRS 2365 (1 μM) had no effect on ICC-DMP Ca^2+^ transient frequency (*P* = 0.25), amplitude (*P* = 0.62), duration (*P* = 0.62), or spatial spread (*P* = 0.4, Figures 8C,D, *n* = 3, *c* = 5). Hyperpolarization of the SIP syncytium with the K_ATP_ channel agonist, pinacidil (10 μM; Figure 9A), also had no effect on ICC-DMP Ca^2+^ transient frequency (*P* = 0.47), amplitude (*P* = 0.36), duration (*P* = 0.26), or spatial spread (*P* = 0.6, Figures 9A,B, *n* = 4, *c* = 16). These data suggest that membrane potential transients have no effect on Ca^2+^ tranisents in ICC-DMP, and purines which exert their hyperpolarizing effects on SIP cells other than ICC-DMP do not affect the Ca^2+^ transients in ICC-DMP.

### Nitrergic signaling molecules expression in ICC

**Figure 10:**
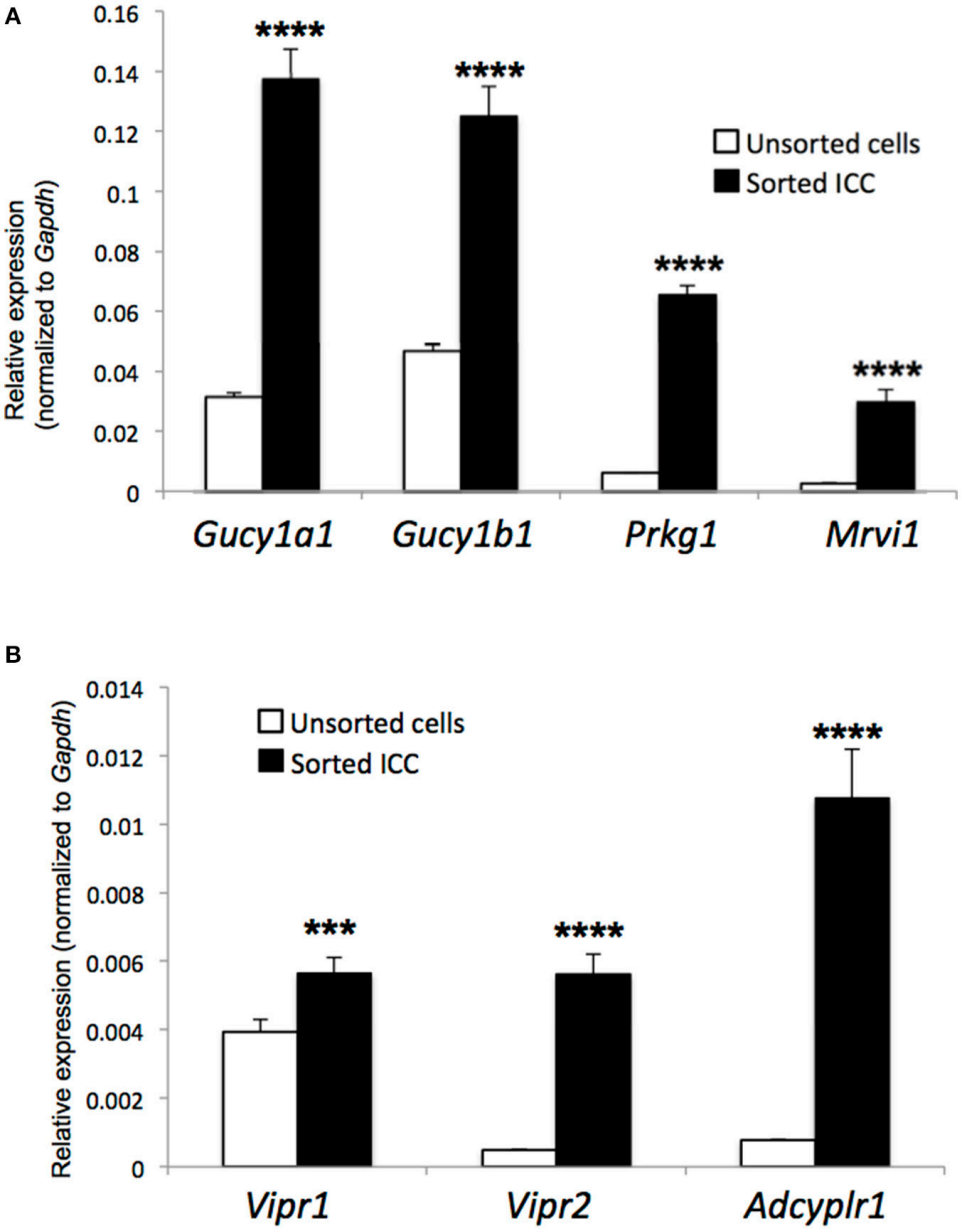
Expression of genes encoding nitrergic and peptidergic signaling molecules in ICC. **(A)** Quantitative PCR (qPCR) data showing the relative expression of transcripts for *Gucy1a1* and *Gucy1b1*, protein kinase cGMP-dependent type 1: *Prkg1*, and inositol-1,4,5 triphosphate receptor I-associated G kinase substrate (IRAG; *Mrvi1*) in sorted small intestinal ICC by FACS and unsorted cells (total cell population). qPCR data is expressed as relative expression, normalized to *Gapdh, n* = 4. **(B)** qPCR data showing the relative expression of transcripts for *Vipr1* and *Vipr2* (VIP receptors) *and Adcyplr1* (PACAP receptor) in FACS sorted small intestinal ICC and unsorted cells (total cell population). qPCR data is expressed as relative expression, normalized to *Gapdh, n* = 4.

As the dominant effects of enteric inhibitory neurotransmission on ICC-DMP appear to depend upon nitrergic stimulation. We examined the expression profile of NO targets in FACS sorted ICC from enzymatic dispersions of small intestinal muscles, as previously described (Baker et al., 2016), and characterized expression of guanylate cyclase 1 soluble subunits alpha 1 and beta 3: *Gucy1a1* and *Gucy1b1*, protein kinase cGMP-dependent type 1: *Prkg1*, and inositol-1,4,5 triphosphate receptor I-associated G kinase substrate (IRAG) (aka murine retrovirus integration site 1 homolog): *Mrvi1* transcripts. We noted elevated expression in all of these downstream mediators of nitrergic responses in ICC relative to unsorted cells (total cell population). *Gucy1a1* transcripts were higher in ICC in comparison to unsorted cells (*Gucy1a1* in ICC: 0.14 ± 0.01 vs. unsorted cells: 0.03 ± 0.001, *P* = 0.0001, *n* = 4; Figure 10A). *Gucy1b1* was also elevated in ICC: 0.13 ± 0.01 vs. unsorted cells 0.046 ± 0.002 (*P* = 0.0001, *n* = 4; Figure 10), *Prkg1* in ICC: 0.07 ± 0.002; unsorted cells: 0.006 ± 0.0001 (*P* = 0.0001, *n* = 4; Figure 10A) and *Mrvi1* in ICC: 0.03 ± 0.004; unsorted cells: 0.003 ± 0.0001 (*P* = 0.0001, *n* = 4; Figure 10A). Thus, the expression of *Gucy1a1, Gucy1b1, Prkg1, and Irag* transcripts are dominant in ICC in comparison to the other cells in the tunica muscularis of the small intestine, and this suggests that ICC have the machinery to mediate nitrergic transmission.

### Activation of guanylate cyclase causes inhibition of Ca^2+^ transients

**Figure 11:**
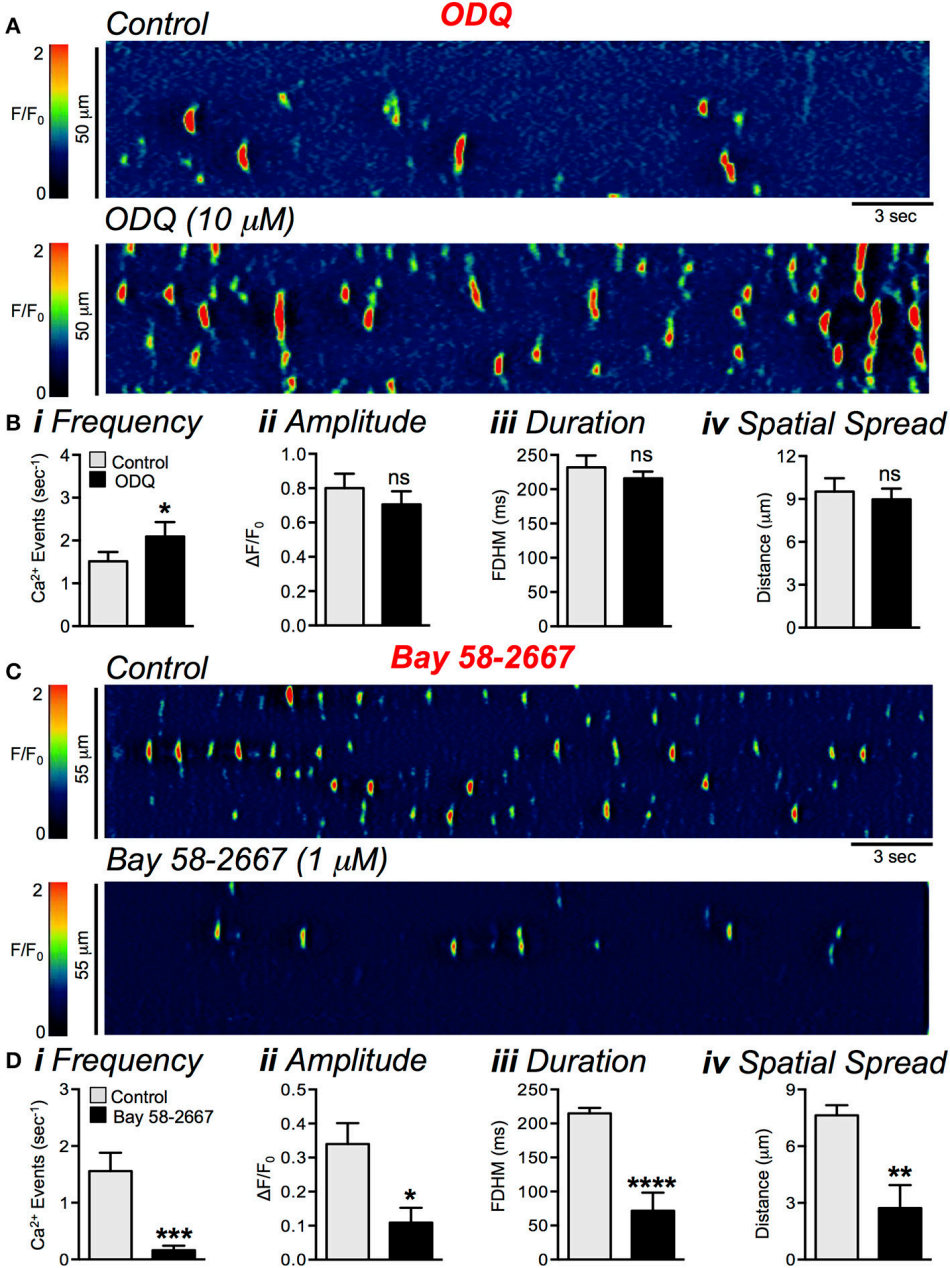
Effects of a soluble guanylyl cyclase (sGC) inhibitor and activator on Ca^2+^ transients in ICC-DMP. **(A)** Representative ST maps showing the effects of sGC inhibitor ODQ (10 μM) on ICC-DMP Ca^2+^ transients. **(B)** Summary graphs showing the effect of ODQ on the frequency **(i)**, amplitude **(ii)**, duration **(iii)**, and spatial spread **(iv)** of spontaneous Ca^2+^ transients in ICC-DMP (*n* = 11, *c* = 25). Note that Ca^2+^ transient events in ICC-DMP were increased in the presence of ODQ. **(C)** Representative ST maps showing the effect of sGC activator Bay 58-2667 (1 μM) on Ca^2+^ transients. **(D)** Summary graphs showing the inhibitory effects of Bay 58-2667 on the frequency **(i)**, amplitude **(ii)**, duration **(iii)**, and spatial spread **(iv)** of Ca^2+^ transients (*n* = 5, *c* = 14). ns = *P* > 0.05, ^*^P < 0.05, ^**^P < 0.01, ^***^P < 0.001, ^****^P < 0.0001.

Similar to L-NNA, ODQ (10 μM), an inhibitor of sGC, significantly increased basal Ca^2+^ transient frequency from 1.5 ± 0.2 events s^−1^ to 2.1 ± 0.3 events sec^−1^ (Figure 11Bi, *P* = 0.03, *n* = 11, *c* = 25; Figures 11A,Bi). The amplitude (*P* = 0.35), duration (*P* = 0.33), and spatial spread (*P* = 0.5) were not significantly different after ODQ (Figures 11Bi–iv, *n* = 7, *c* = 13). The role of sGC in mediating nitrergic responses in ICC-DMP was also shown by an activator of sGC. Bay 58-2667 (1 μM), reduced firing frequency of Ca^2+^ transients (Figures 11C,D) from 1.6 ± 0.3 to 0.2 ± 0.08 s^−1^ (Figure 11Di, *P* = 0.0004, *n* = 5, *c* = 14), amplitude from 0.3 ± 0.06 ΔF/F_0_ to 0.1 ± 0.04 ΔF/F_0_ (Figure 11Dii, *P* = 0.02, *n* = 5, *c* = 14), duration from 215 ± 8.2 ms to 72 ± 26.9 ms (Figure 11Diii, *P* = 0.0001, *n* = 5, *c* = 14), and spatial spread from 7.6 ± 0.5 μm to 2.7 ± 1.2 μm (Figure 11Div, *P* = 0.0019, *n* = 5, *c* = 14).

**Figure 12:**
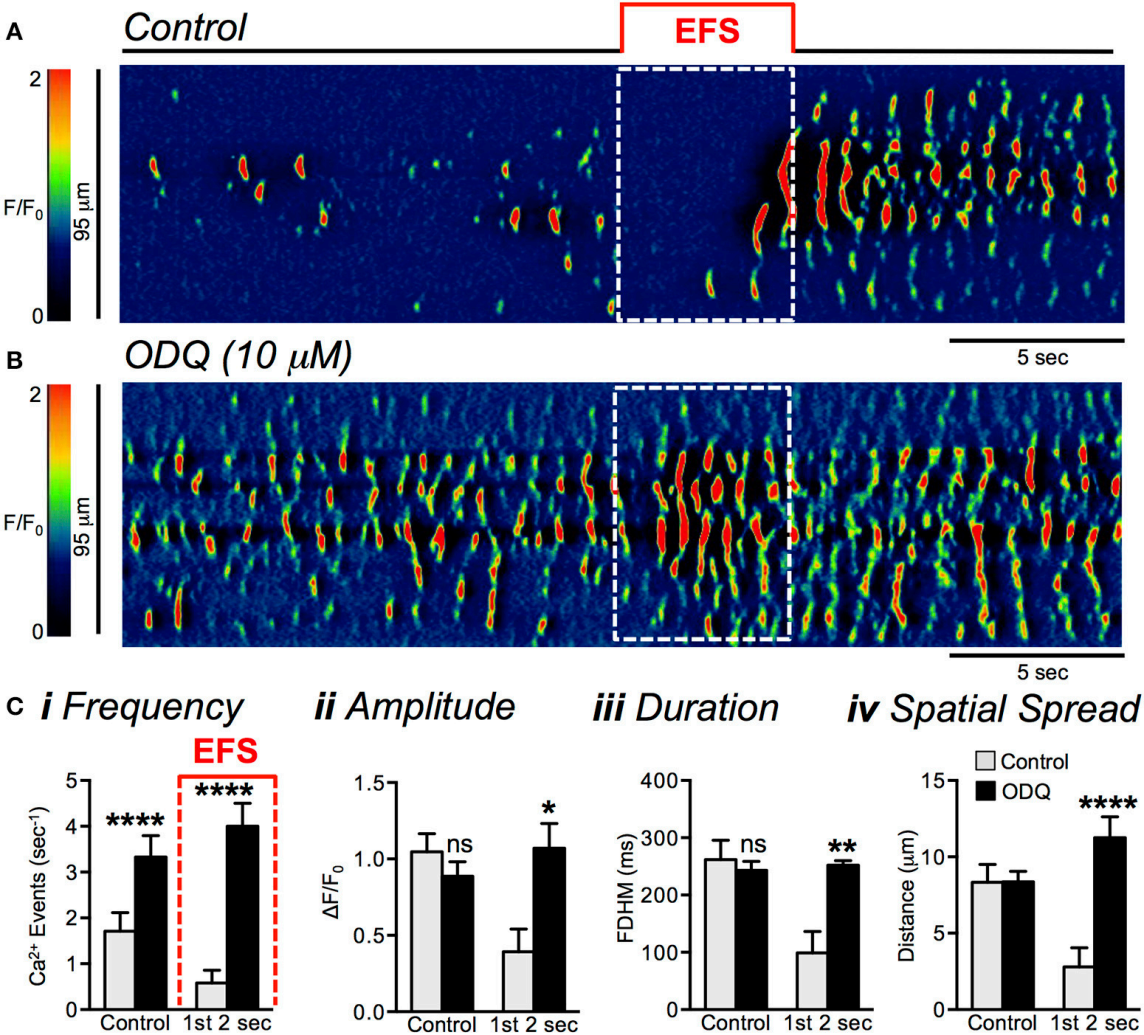
Effects of soluble guanylyl cyclase (sGC) inhibitor (ODQ) on EFS-evoked inhibitory Ca^2+^ responses in ICC-DMP. **(A,B)** Representative ST maps showing Ca^2+^ transient events inhibition in response to nerve stimulation (EFS at 10 Hz for 5 s; indicated by the red line and dotted white box in ST maps) as shown in **(A)**. EFS failed to induce inhibitory effects on Ca^2+^ transients In the presence of ODQ (10 μM) as shown in **(B)**. **(C)** Summary data showing the effects of ODQ (10 μM) on Ca^2+^ transient frequency **(i)**, amplitude **(ii)**, duration **(iii)**, and spatial spread **(iv)** in ICC-DMP during control conditions (pre-EFS), and in the 1st 2 s of EFS and in the presence of ODQ (*n* = 3, *c* = 12). ns = *P* > 0.05, ^*^P < 0.05, ^**^P < 0.01, ^****^P < 0.0001.

ODQ (10 μM) also blocked the inhibitory effects of EFS on ICC-DMP during the initial 2 s of stimulation (Figures 12A–C). In these experiments the frequency of Ca^2+^ transients during the first 2 s of EFS was 0.6 ± 0.3 s^−1^ in control and increased to 4 ± 0.5 s^−1^ in the presence of ODQ (Figure 12Ci, *P* < 0.0001, *n* = 5, *c* = 25). ODQ also increased Ca^2+^ transient amplitude during EFS from 0.4 ± 0.15 ΔF/F_0_ to 1.1 ± 0.2 ΔF/F_0_ (Figure 12Cii, *P* = 0.02, *n* = 5, *c* = 25), duration from 99 ± 37.2 ms to 252 ± 7.8 ms (Figure 12Ciii, *P* = 0.003, *n* = 5, *c* = 25), and spatial spread from 2.8 ± 1.3 μm to 11.3 ± 1.4 μm (Figure 12Civ, *P* < 0.0001, *n* = 5, *c* = 25).

### Pharmacological inhibitors of PKG failed to modulate ICC-DMP Ca^2+^ transients

**Figure 13:**
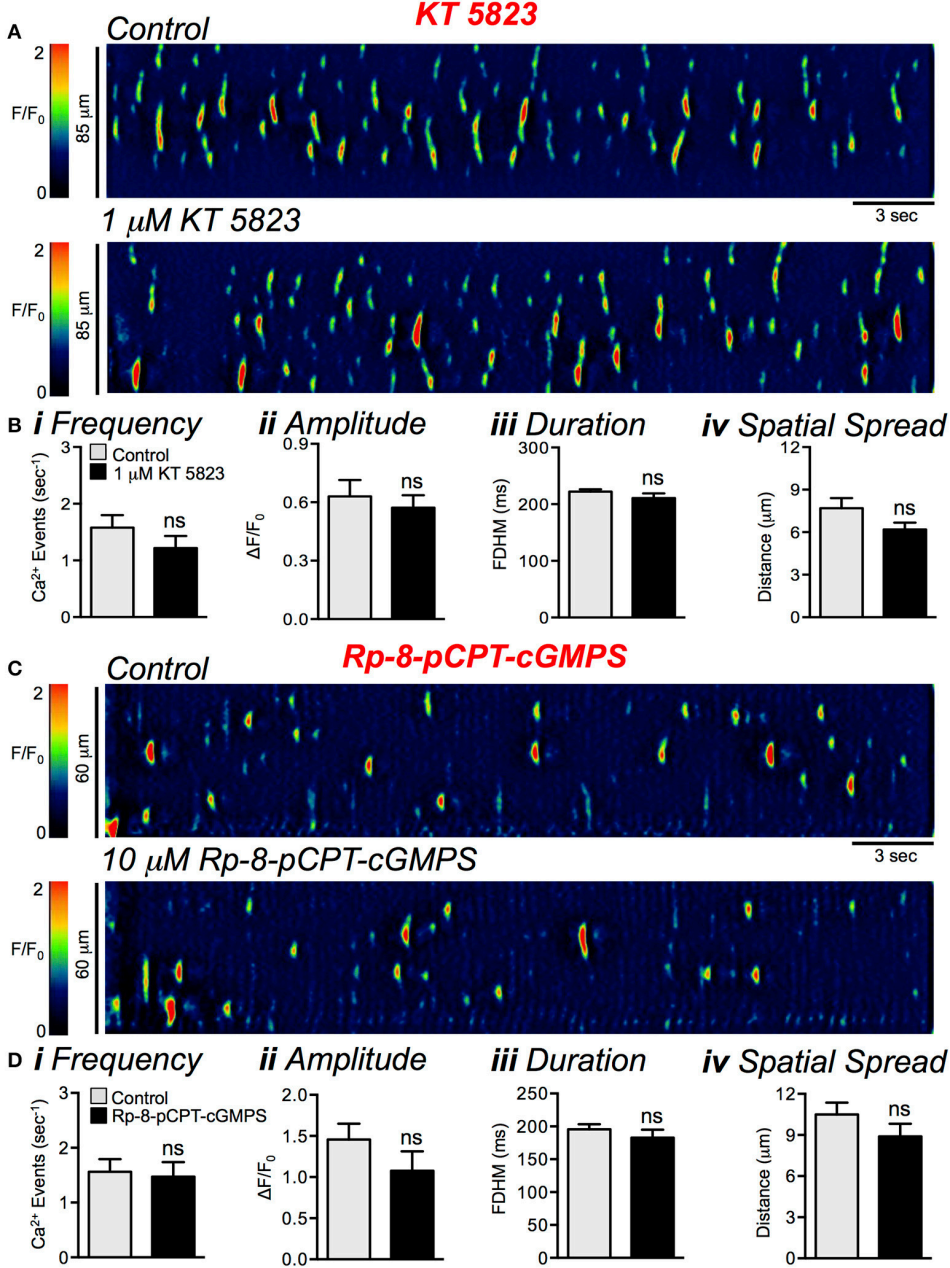
PKG inhibitors had no effect on basal Ca^2+^ transients in ICC-DMP. **(A)** Representative ST maps showing the effect of KT 5823 (1 μM) on Ca^2+^ spontaneous transients in ICC-DMP. **(B)** Summary graphs showing the lack of effect of KT 5823 on the frequency **(i)**, amplitude **(ii)**, duration **(iii)**, and spatial spread **(iv)** of basal Ca^2+^ transients in ICC-DMP (*n* = 4, *c* = 11). **(C)** Representative ST maps showing the lack of effect of Rp-8-pCPT-cGMPS (10 μM) on basal Ca^2+^ transients in ICC-DMP. **(D)** Summary graphs showing the lack of effect of Rp-8-pCPT-cGMPS on the frequency **(i)**, amplitude **(ii)**, duration **(iii)**, and spatial spread **(iv)** of Ca^2+^ transients in ICC-DMP (*n* = 8, *c* = 21). ns = *P* > 0.05.

We next sought to evaluate the role of cGMP dependent protein kinase (PKG) on basal firing of Ca^2+^ transients in ICC-DMP. We tested the effects of two PKG inhibitors, KT 5823 (1 μM) and Rp-8-pCPT-cGMPS (10 μM). However, Neither of these compounds had any effect on the basal activity of Ca^2+^ transients in ICC-DMP (Figures 13A–D). KT 5823 failed to significantly affect Ca^2+^ transient frequency (Figure 13Bi; *P* = 0.08), amplitude (Figure 13Bii; *P* = 0.58), duration (Figure 13Biii; *P* = 0.14) or spatial spread (Figure 13Biv; *P* = 0.09), *n* = 4, *c* = 11. Rp-8-pCPT-cGMPS also failed to exert any significant changes in Ca^2+^ transient frequency (Figure 13Di; *P* = 0.65), amplitude (Figure 13Dii; *P* = 0.14), duration (Figure 13Diii; *P* = 0.19) or spatial spread (Figure 13Div; *P* = 0.19), *n* = 8, *c* = 21. We next tested if inhibition of PKG relieved the EFS evoked inhibitory response in ICC-DMP. KT 5823 and Rp-8-pCPT-cGMPS both had no significant effects on Ca^2+^ transients during the initial 2 s period of EFS. Across all cells tested, there was no significant change in the firing frequency (*P* = 0.57), amplitude (*P* = 0.49), duration (0.96) or spatial spread (0.58) of Ca^2+^ transients during the initial 2 s period of EFS in the presence of KT 5823 (data not shown, *n* = 3, *c* = 6). Also in the presence of Rp-8-pCPT-cGMPS (10 μM) there was no significant change in the firing frequency (*P* = 0.67), amplitude (*P* = 0.37), duration (0.92) or spatial spread (0.81) of Ca^2+^ transients during the initial 2 s period of EFS in the presence of the drug (data not shown, *n* = 5, *c* = 11).

We also tested the PKG inhibitors seeking a positive control for their poor performance against nitrergic effects in ICC. Contractile experiments were performed on muscles of the colon, aorta and corpus cavernosum. Neither KT 5823 nor Rp-8-pCPT-cGMPS reduced the inhibitory effects of sodium nitroprusside (SNP; 100 nM) or DEA NONOate (10 μM) on muscles pre-contracted with norepinephrine (NE; 100 nM) or carbachol (CCh; 10 μM) (data not shown). Taken together, the lack of effects of commercial PKG inhibitors on smooth muscles, in general, might be due to poor penetration of the drugs into cells in intact tissues or targets besides PKG contributing to inhibitory responses. This is a question in need of further evaluation.

### Vasoactive intestinal peptide (VIP) modulation of ICC-DMP Ca^2+^ transients

Nitrergic modulation of ICC-DMP transients is an important aspect of inhibitory neurotransmission, but neuropeptides (VIP and PACAP: pituitary adenylate cyclase-activating peptide) are also released from nerve terminals and might modulate ICC-DMP activity. Therefore, we examined the expression profile of peptide receptors in sorted ICC from small intestinal muscles and characterized expression of VIP receptors (*Vipr1* and *Vipr2*) and PACAP receptor (*Adcyap1r1)*. We noted elevated expression in all of peptidergic receptors in ICC relative to unsorted cells (total cell population). *Vipr1* transcripts were higher in ICC in comparison to unsorted cells (*Vipr1* in ICC: 0.006 ± 0.0005 vs. unsorted cells: 0.004 ± 0.0004, *P* = 0.001, *n* = 4; Figure 10B). Also *Vipr2* in ICC: 0.006 ± 0.0006 vs. unsorted cells 0.0005 ± 0.00001 (*P* = 0.0001, *n* = 4; Figure 10B) and *Adcyap1r1* in ICC: 0.01 ± 0.001; unsorted cells: 0.0008 ± 0.00001 (*P* = 0.0001, *n* = 4; Figure 10B). Peptidergic receptors are abundant in ICC suggesting that ICC has the machinery to mediate inhibitory peptidergic transmission.

**Figure 14:**
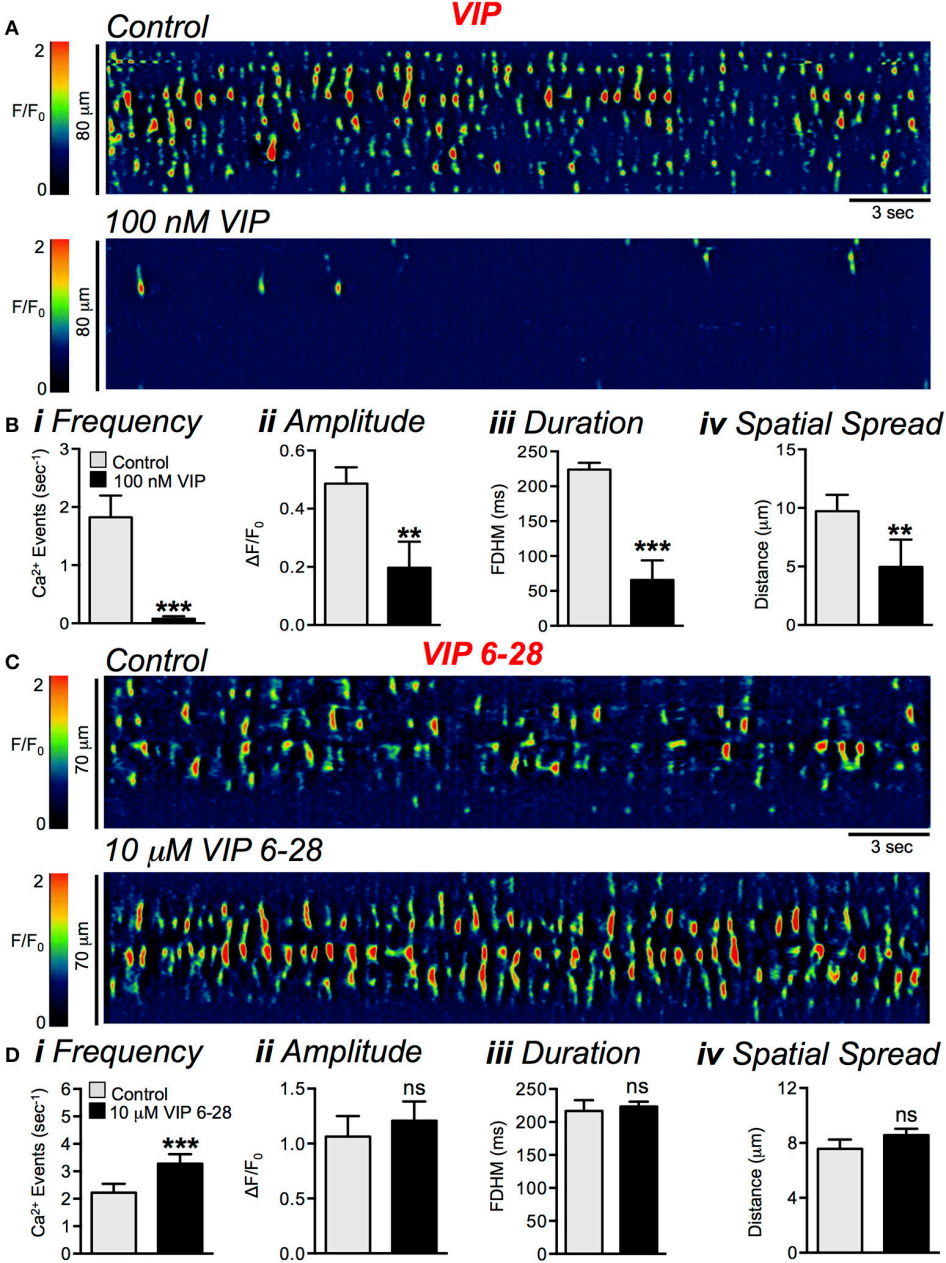
VIP modulation of spontaneous ICC-DMP Ca^2+^ transients. **(A)** Representative ST maps showing the effects of VIP (100 nM) on Ca^2+^ transients in ICC-DMP. **(B)** Summary graphs showing the effect of VIP on the frequency **(i)**, amplitude **(ii)**, duration **(iii)**, and spatial spread **(iv)** of Ca^2+^ transients in ICC-DMP (*n* = 5, *c* = 12). **(C)** Representative ST maps showing the effect of VIP 6-28 (10 μM) on basal Ca^2+^ transients in ICC-DMP. **(D)** Summary graphs showing the effect of VIP 6–28 on the frequency **(i)**, amplitude **(ii)**, duration **(iii)**, and spatial spread **(iv)** of Ca^2+^ transients in ICC-DMP (*n* = 5, *c* = 23). ns = *P* > 0.05, ^**^P < 0.01, ^***^P < 0.001.

We also tested the effects of VIP sensitive inputs to ICC-DMP. As shown in Figure 14, application of VIP (100 nM) led to inhibition of Ca^2+^ transient firing in ICC-DMP (Figure 14A). VIP reduced the firing frequency of Ca^2+^ transients from 1.8 ± 0.4 s^−1^ in control to 0.08 ± 0.04 s^−1^ (Figure 14Bi, *P* = 0.0004, *n* = 5, *c* = 12). Similarly, the amplitude of Ca^2+^ transients was reduced from 0.5 ± 0.06 ΔF/F_0_ to 0.2 ± 0.08 ΔF/F_0_ by VIP (Figure 14Bii, *P* = 0.006, *n* = 5, *c* = 12). The duration of Ca^2+^ transients was decreased from 224 ± 9.8 ms to 66 ± 28 ms by VIP (Figure 14Biii, *P* = 0.0001, *n* = 5, *c* = 12). Finally, VIP also significantly decreased the spatial spread of Ca^2+^ transients from 9.7 ± 1.4 μm to 5 ± 2.3 μm (Figure 14Biv, *P* = 0.003, *n* = 5, *c* = 12). VIP 6-28 (10 μM) caused a significant increase in basal Ca^2+^ transient firing in a similar manner to blocking nitrergic input with application of L-NNA (Figure 14C). VIP 6-28 increased basal Ca^2+^ transient firing from 2.2 ± 0.3 to 3.3 ± 0.3 s^−1^ (Figure 14Di, *P* = 0.0007, *n* = 5, *c* = 23). The amplitude (*P* = 0.38), duration (*P* = 0.67) and spatial spread (*P* = 0.24) of Ca^2+^ transients was not significantly affected by VIP 6-28, *n* = 5, *c* = 23 (Figure 14D).

**Figure 15:**
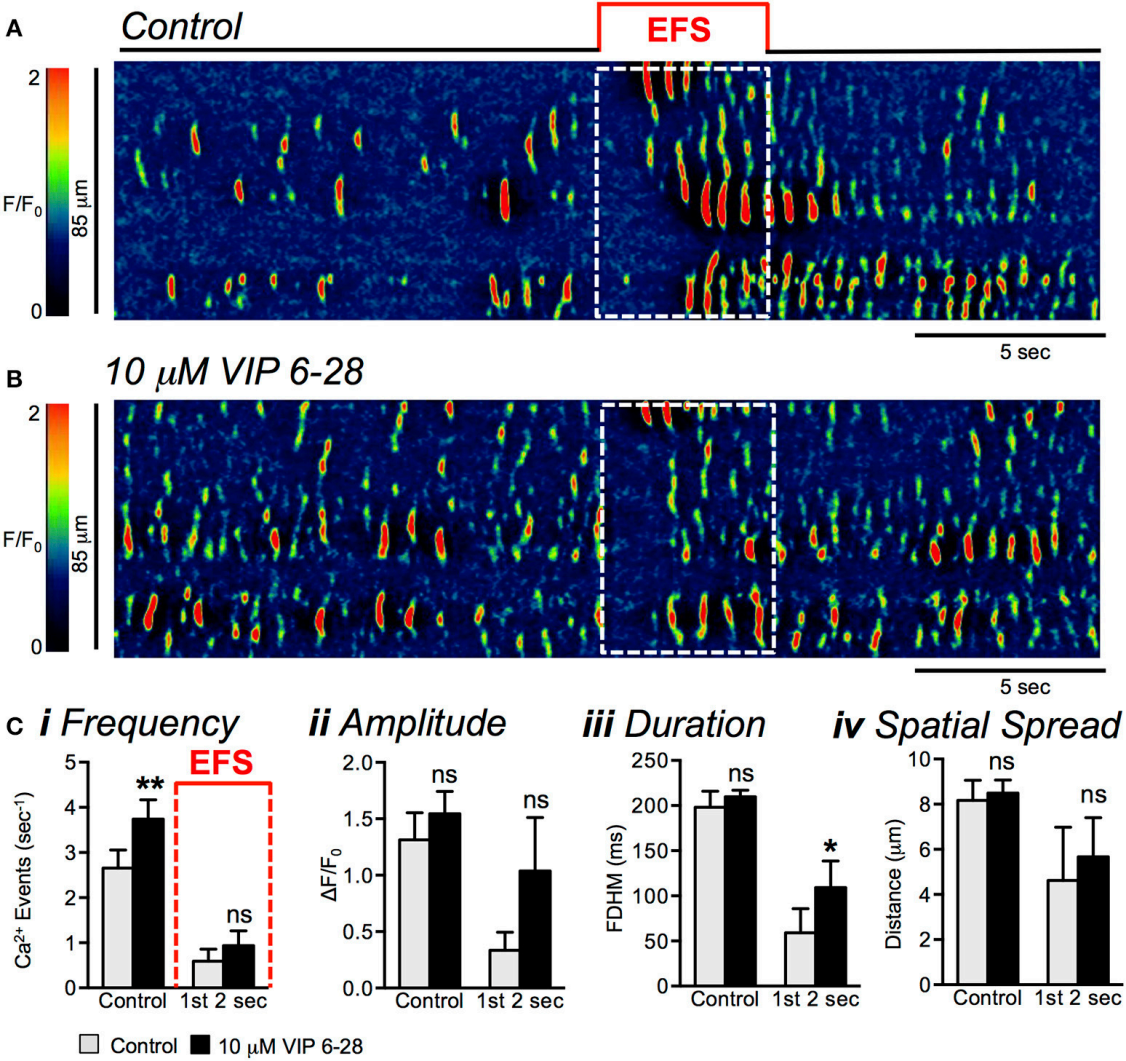
VIP 6-28 failed to block inhibitory responses in ICC-DMP to EFS. **(A,B)** Representative ST maps showing the effects of VIP 6-28 (10 μM) on Ca^2+^ transients in response to EFS (10 Hz 5 s; indicated by the red line and dotted white box in ST maps). **(C)** Summary data showing the inhibitory effects of VIP 6-28 (10 μM) on Ca^2+^ transient frequency **(i)**, amplitude **(ii)**, duration **(iii)**, and spatial spread **(iv)** in ICC-DMP during control conditions (pre-EFS), and in the 1st 2 s of EFS (*n* = 5, *c* = 16). ns = *P* > 0.05, ^*^P < 0.05, ^**^P < 0.01.

While VIP 6-28 increased the basal level of Ca^2+^ transient firing in a similar manner to L-NNA (Figure 14), VIP 6-28 did not significantly relieve EFS-evoked inhibitory responses (Figures 15A–C). Neither the frequency (*P* = 0.11), amplitude (*P* = 0.16), or spatial spread (*P* = 0.58) of Ca^2+^ transients was significantly affected during the initial 2 s period of EFS VIP 6–28 (10 μM) (Figure 15C, *n* = 5, *c* = 16). However, the duration of Ca^2+^ transients in the initial 2 s period of EFS was significantly increased from 59 ± 26.9 ms in control to 110 ± 29.5 ms in the presence of VIP 6-28 (Figure 15Ciii, *P* = 0.023, *n* = 5, *c* = 16). It is possible that multiple receptors mediate responses to inhibitory peptides in ICC.

## Discussion

In this study we examined enteric inhibitory regulation of Ca^2+^ transients in ICC-DMP, the intramuscular class of ICC in the small intestine. Ca^2+^ handling mechanisms in ICC are of interest because Ca^2+^ release from stores couples to activation of Ano1 channels and the generation of STICs (Zhu et al., 2011, 2015). In the case of ICC-DMP, Ca^2+^ transients have only localized influence within cells, and no evidence for propagation of Ca^2+^ transients between cells, even over long periods of observation, was obtained (Baker et al., 2016). Localized, stochastic events occurring in hundreds or thousands of ICC-DMP could have significant influence on the excitability of cells of the SIP syncytium to which ICC-DMP are electrically coupled by abundant gap junctions (Zhou and Komuro, 1992a; Torihashi et al., 1993; Seki and Komuro, 1998). We found that nitrergic mechanisms are the primary neural inhibitory regulators of Ca^2+^ transients in ICC-DMP, and regulation by purines was not resolved. As in many other cells, nitrergic input was transduced by binding to its natural receptor, sGC, and downstream effects were mediated by cGMP (Bult et al., 1990; Moncada et al., 1991; Pfeifer et al., 1998; Somlyo and Somlyo, 2003). Our observation, linking cGMP to inhibition of Ca^2+^ release events in ICC-DMP, is a novel aspect of nitrergic regulation, and as discussed below, this is likely to be one of the fundamental inhibitory mechanisms of nitrergic regulation in GI motility. We also provide novel evidence that peptidergic neurotransmission is superimposed on ICC-DMP and provides a portion of tonic inhibition in ICC-DMP.

Interstitial cells, ICC and PDGFRα^+^ cells, are functional elements of the SIP syncytium that regulate the excitability of SMCs in all smooth muscle regions of the GI tract (Sanders et al., 2014b). Interstitial cells transduce different parts of the motor neural inputs that regulate GI motility (Burns et al., 1996; Ward et al., 2000; Iino et al., 2004; Kurahashi et al., 2011; Baker et al., 2015). A role for ICC in neurotransmission was suggested from morphological studies that described close associations between varicose nerve terminals and ICC (Rumessen et al., 1992; Zhou and Komuro, 1992b; Faussone-Pellegrini, 2006; Blair et al., 2012). Studies in animals lacking intramuscular ICC demonstrated that these cells contribute to post-junctional responses to both excitatory and inhibitory neurotransmission (Burns et al., 1996; Wang et al., 2003a; Iino et al., 2004; Klein et al., 2013; Sanders et al., 2014a) and cell specific knock down of sGC in ICC (Groneberg et al., 2015) have also been consistent with a role for ICC in neurotransmission. In the small intestine, inhibitory and excitatory post-junctional neural responses follow age-dependent development of ICC-DMP (Ward et al., 2006), and blocking Kit with neutralizing c-Kit antibody caused reduction in ICC-DMP and loss of cholinergic and nitrergic neural responses. These previous findings are in agreement with the results of the present study: (i) ICC-DMP have the necessary molecular machinery to transduce signals arising from motor neurons; (ii) ICC-DMP are innervated by enteric inhibitory motor neurons; (iii) Inhibitory neurotransmission regulates the occurrence of Ca^2+^ transients in ICC-DMP, thus controlling Ca^2+^ transients necessary for activation of electrophysiological responses in the SIP syncytium (Zhu et al., 2011, 2015).

We focused on neuromodulation of Ca^2+^ transients in ICC-DMP because Ca^2+^ transients are coupled to activation of Ano1 channels and STICs in these cells (Zhu et al., 2011, 2015). Thus, regulation of Ca^2+^ transients, which are ongoing in these cells, provides a means of bi-directional regulation of excitability of SMCs. Turning STICs off reduces net inward current in the SIP syncytium, and such a signal would favor stabilization of excitability; increasing STICs increases net inward current in the SIP syncytium and adds a depolarizing influence that increases the excitability of SMCs. In a previous study, we showed that inhibition of basal nerve activity with TTX increases Ca^2+^ transients (Baker et al., 2016). In the present study we demonstrated that inhibition of NO synthesis with L-NNA, or inhibition of cGMP synthesis with ODQ also increased Ca^2+^ transients. This would lead to increased inward current and at least partial blockade of what has been termed “tonic inhibition” of GI muscles (Wood, 1972). Inhibitory peptides also appear to contribute to tonic inhibition because Ca^2+^ transients were increased by VIP 6-28. We also found that stimulation of intrinsic neurons by EFS caused a brief period of inhibition (~2 s) in which ongoing Ca^2+^ transients in ICC-DMP were largely abolished. Shutting off of STICs during the initial phase of EFS may explain a portion of the hyperpolarization responses to nerve stimulation seen in electrophysiological recordings (Stark et al., 1991).

It is difficult to reconcile generalized tissue level responses to EFS (e.g., electrophysiological events and contractions) with events occurring in a single type of ICC. Neurotransmission may affect conductances and Ca^2+^ sensitization mechanisms in multiple cells (SMCs, PDGFRα^+^ cells, and in other types of ICC) leading to non-linear changes in voltage-dependent conductances, membrane potential and excitation-contraction coupling. At present there are no organ-specific, unified models of the many responses in GI muscles that might be initiated or repressed by stimulation of enteric neurons.

During 5 s periods of EFS, Ca^2+^ firing sites within ICC-DMP escaped from inhibition. In our experiments this occurred within about 2 s from the onset of stimulation. However, the Ca^2+^ release sites escaped inhibition with different temporal characteristics. The heterogeneity in the periods before escape from inhibition occurred is further demonstration of the independence of Ca^2+^ release sites, even within individual cells, and supports the conclusion that there is no cellular or multi-cellular correlation between Ca^2+^ release sites within the ICC-DMP network (Baker et al., 2016). The variability in the period before escape from inhibition might be attributed to several factors. There may be variability in the size or molecular composition of different release sites (e.g., relative balance between RyRs and IP_3_Rs or relative density or distribution of SERCA pumps). We showed that Ca^2+^ release events were virtually blocked if neurokinin receptor antagonists were present (Baker et al., 2018). Thus, it was not possible to investigate inhibitory responses in isolation of excitatory neural inputs. Therefore, another factor affecting the escape from inhibition might be the relative density of excitatory varicosities and distribution of post-junctional receptors and/or effectors along the lengths of ICC-DMP. Greater excitatory or inhibitory neural inputs at a given site might accelerate or delay the escape from inhibition. Likewise, a greater concentration of post-junctional inhibitory or excitatory pathway components may impact the rate of escape.

Soluble guanylyl cyclase (sGC) is expressed in ICC and is the main receptor/transducer of the inhibitory effects of NO in the GI tract (Shuttleworth et al., 1993; Salmhofer et al., 2001; Iino et al., 2008, 2009; Cobine et al., 2014; Lies et al., 2014, 2015; Sanders, 2016). Previous immunohistochemical studies have shown that sGC-α and sGC-β are both expressed in ICC of lower esophageal sphincter, stomach, small intestine, caecum, colon, and internal anal sphincter (Salmhofer et al., 2001; Iino et al., 2009; Cobine et al., 2014; Lies et al., 2014, 2015), and at least from immunohistochemical analyses, sGC is more abundant in ICC than in SMCs. We confirmed these findings in the small intestine and found high expression of *Gucy1a1* and *Gucy1b1* in sorted ICC relative to the unsorted cell population (which would contain SMCs). Thus, ICC have the receptor and the molecular apparatus to transduce nitrergic signals and produce cGMP. Others have found that knock down of *Gucy1b1* in ICC, using Cre-LoxP technology, abolished nitrergic inhibitory junction potentials (IJP) in gastric fundus and reduced IJPs in the colon, but these authors also reported that knockdown of *Gucy1b1* in SMCs also either reduced the amplitude or shortened IJPs (Lies et al., 2014). Another study concluded that nitrergic relaxation of fundus muscles depends upon sGC in both ICC and SMCs (Groneberg et al., 2013). We found that nitrergic inhibition of Ca^2+^ transients in ICC-DMP depends upon the sGC, as inhibitors and activators of sGC effectively modulated Ca^2+^ release.

How cGMP regulates Ca^2+^ release in ICC is complicated. The traditional view is that cGMP-dependent protein kinase-1 (PKG1; encoded by *Prkg1*) is the principal downstream signaling molecule mediating nitrergic responses, and *Prkg1* is expressed in ICC in the small intestine (Salmhofer et al., 2001), as also confirmed by the present study. cGMP is thought to activate PKG1 and cause phosphorylation of downstream targets (Xue et al., 2000; Hofmann, 2005). These targets in ICC have not been defined precisely. However, one study showed that cell-specific knockdown of *Prkg1* in ICC reduced NO-dependent inhibitory junction potentials in colonic smooth muscles (Klein et al., 2013). A signaling molecule downstream of PKG1 appears to be inositol triphosphate receptor (IP_3_R)-associated cGMP-kinase substrate (IRAG; encoded by *Mrvi1*), and this gene is also expressed in ICC of the small intestine. IRAG co-precipitates with IP_3_R and was found to be indispensable for cGMP regulation of Ca^2+^ release in model cells or cultured human colonic SMCs (Schlossmann et al., 2000; Fritsch et al., 2004). IRAG is phosphorylated by PKG1β at Ser696 and suppresses Ca^2+^ release from IP_3_R1 (Masuda et al., 2010; Schlossmann and Desch, 2011), and transcripts of the PKG1β splice variant (NM_ 011160) were 23-fold more abundant than transcripts of the PKG1α variant (NM_001013833) from RNA-seq of small intestinal ICC (Lee et al., 2017). As shown in the present study by real-time PCR, all of these signaling molecules are present in small intestinal ICC and more strongly expressed in ICC than in the general population of cells dispersed from the *tunica muscularis* of the jejunum. Therefore, this pathway might represent the primary mechanism for nitrergic suppression of Ca^2+^ transients and waves in ICC. However, a recent paper showed that nitrergic relaxation was only slightly reduced in murine internal anal sphincter muscles of PKG^−/−^ mice, and L-NNA abolished relaxations to nitrergic nerve stimulation in both wildtype and PKG^−/−^ mice (Cobine et al., 2014). These findings suggest that significant cGMP-dependent, but PKG independent, pathways may contribute to nitrergic responses, and pathways specific to ICC will require additional investigation. In the present study PKG inhibitors had no effect on nitrergic responses, but these drugs appear to have penetration problems in whole muscles because they also failed to block nitrergic responses in several smooth muscle preparations (colon, aorta, and corpus cavernosum). PKG inhibitors also failed to have any significant effects on ICC pacemaker potentials (Koh et al., 2000; Shahi et al., 2014). Taken together, PKG inhibitors do not appear to be suitable for *in situ* studies, and genetic models with combinations of deleted genes and expression of optogenetic sensors appear to be needed for future studies to address downstream signaling mechanisms responsible for neural regulation of ICC.

The lack of purinergic effects on Ca^2+^ transients in ICC-DMP might seem surprising since it is well-known that purines contribute significantly to enteric inhibitory regulation of GI muscles (Burnstock et al., 1970; Gallego et al., 2014; Jimenez et al., 2014; Sanders, 2016). P2Y1 receptors mediate purinergic enteric neural inhibition in GI muscles, as shown by pharmacological and gene deactivation studies (Gallego et al., 2012, 2014; Hwang et al., 2012; Gil et al., 2013). However, dominant expression of *P2ry1* is found in PDGFRα^+^ interstitial cells, not SMCs or ICC, and purinergic inhibitory effects are mediated through PDGFRα^+^ cells (Kurahashi et al., 2011, 2014; Baker et al., 2013, 2015). P2Y1 receptor agonists hyperpolarize PDGFRα^+^ cells by activation of small conductance, Ca^2+^ activated K^+^ channels, and hyperpolarization responses are conducted to other SIP cells (Kito et al., 2014). Thus, the lack of purinergic effects on ICC is compensated by effects of purines on PDGFRα^+^ cells.

Pinacidil, through activation of K_ATP_ in GI SMCs, also causes hyperpolarization of the SIP syncytium (Koh et al., 1998; Kito et al., 2005), but this agonist had no effect on Ca^2+^ transients in ICC-DMP. Likewise, hyperpolarization of PDGFRα^+^ cells by the P2Y1 specific agonist MRS2365 (which has no effect on GI muscles lacking P2Y1 receptors; Hwang et al., 2012); also had no effect on Ca^2+^ transients in ICC-DMP. These data demonstrate that substances that cause openings of K^+^ channels and exert hyperpolarizing effects on the SIP syncytium, do not interfere with the Ca^2+^ release events occurring in the ICC-DMP component of the syncytium. We also know from previous studies that depolarization does not affect Ca^2+^ transients in ICC-DMP. Our imaging studies were conducted on full thickness jejunal muscles that undergo periodic depolarizations from slow wave activity; yet there is no periodic behavior in the firing of Ca^2+^ transients that might indicate regulation of Ca^2+^ release by a voltage-dependent mechanism (Baker et al., 2016). In fact our data illustrate an important design feature of the SIP syncytium: By lacking a voltage-dependent mechanism that coordinates Ca^2+^ release events, ICC-DMP are protected from the effects of compounds having membrane potential effects in other SIP cells. This allows neural regulation of ICC-DMP without having this mechanism pre-activated or deactivated by events occurring in other cells.

Peptidergic inhibitory neurotransmission also contributes to regulation of motility in the small intestine (Ekblad et al., 2000; Lazar et al., 2001; Matsuyama et al., 2002; Sanders, 2016). In the present study we found that Ca^2+^ transients in ICC-DMP are also regulated by inhibitory peptides. VIP 6-28 enhanced basal Ca^2+^ transient activity, suggesting ongoing release of peptidergic neurotransmitters and contributions from peptides to tonic inhibition. Neurotransmission involving inhibitory peptides during EFS is more complicated and may involve binding of transmitters to multiple post-junctional receptors, as several are expressed and VIP6-28 failed to block neural responses.

In summary, Ca^2+^ transients in ICC-DMP are suppressed under basal conditions by TTX and this appears to occur by blocking release of NO and inhibitory peptides from intrinsic neurons. EFS caused inhibition of Ca^2+^ transients, but ICC-DMP escaped from inhibition during 5 s trains of stimuli. The inhibitory period was due maingly to nitrergic effects mediated by cGMP. Purinergic inputs, that occur in parallel to release of NO in the GI muscles, did not affect Ca^2+^ transients in ICC-DMP and agonists that hyperpolarize other cells in the SIP syncytium also were ineffective in modulating Ca^2+^ transients in ICC-DMP. These data demonstrate a lack of voltage-dependent regulation of Ca^2+^ transients in ICC-DMP. Peptidergic neurotransmission can also modulate Ca^2+^ ICC-DMP, but the receptor(s) responsible for these effects are complex. Ca^2+^ transients initiate inward currents in ICC-DMP that are conducted to other cells in the SIP syncytium. Thus, suppression of Ca^2+^ transients in ICC-DMP by inhibitory neural inputs would tend to reduce SMC excitability and reduce contractile force in the small intestine.

## Author contributions

SB, BD, and KS: Conception and design of the experiments; SB, BD, CC, KK, and KS: Collection, analysis, and interpretation of data; SB, BD, and KS: Drafting the article and revising it critically for intellectual content. All authors read and approved the manuscript before submission.

### Conflict of interest statement

The authors declare that the research was conducted in the absence of any commercial or financial relationships that could be construed as a potential conflict of interest. The reviewer MC and handling Editor declared their shared affiliation.

## Abbreviations

CM: Circular Muscle
FOV: Field of view
GC: Guanylate cyclase
GI: Gastrointestinal
ICC: Interstitial Cells of Cajal
ICC-DMP: Interstitial cells of Cajal at the level of the deep muscular plexus
ICC-IM: Intramuscular interstitial cells of Cajal
GCaMP3: Genetically encoded Ca^2+^ indicator composed of a single GFP
IP_3_: Inositol 1,4,5-trisphosphate
InsP_3_R: Inositol triphosphate receptor
KRB: Krebs Ringer Bicarbonate
LM: Longitudinal Muscle
NO: Nitric oxide
PACAP: Pituitary adenylate cyclase-activating peptide
PDGFRα: Platelet derived growth factor receptor α
ROI: Region of interest
RyR: Ryanodine receptor
SERCA: Sarco/endoplasmic reticulum Ca^2+^-ATPase
sGC: Soluble guanylate cyclase
SIP syncytium: Electrical syncytium formed by Smooth muscle cells; ICC and PDGFRα^+^, cells in GI muscles
STIC: Spontaneous transient inward current
VIP: Vasoactive intestinal peptide.
